# Obesity‐Associated Adiposomes Promote Vascular Smooth Muscle Cell Hypercontractility

**DOI:** 10.1002/cph4.70053

**Published:** 2025-09-18

**Authors:** Elsayed Metwally, Imaduddin Mirza, Mohammed H. Morsy, Sarah M. Mostafa, Francesco M. Bianco, Chandra Hassan, Mario A. Masrur, Paola C. Rosas, Irena Levitan, Usha J. Raj, Brian T. Layden, Abeer M. Mahmoud

**Affiliations:** ^1^ Department of Medicine, Division of Endocrinology, Diabetes, and Metabolism, College of Medicine University of Illinois Chicago Chicago Illinois USA; ^2^ Department of Cytology and Histology, Faculty of Veterinary Medicine Suez Canal University Ismailia Egypt; ^3^ Department of Pharmacology, College of Medicine University of Illinois at Chicago Chicago Illinois USA; ^4^ Department of Surgery, College of Medicine University of Illinois Chicago Chicago Illinois USA; ^5^ Department of Pharmacy Practice, College of Pharmacy University of Illinois at Chicago Chicago Illinois USA; ^6^ Division of Pulmonary and Critical Care Medicine, Department of Medicine University of Illinois at Chicago Chicago Illinois USA; ^7^ Department of Pediatrics, College of Medicine University of Illinois Chicago Chicago Illinois USA; ^8^ Jesse Brown Veterans Affairs Medical Center Chicago Illinois USA; ^9^ Department of Kinesiology and Nutrition, College of Applied Health Sciences University of Illinois Chicago Chicago Illinois USA

**Keywords:** adiposomes, Ca^2+^ signaling, ceramide, hypercontractility, K_ATP_ channels, metabolic syndrome, obesity, reactive oxygen species, vascular dysfunction, vascular smooth muscle cells

## Abstract

**Background:**

Inter‐organ crosstalk, particularly between adipose tissue and vasculature, plays a key role in obesity‐induced cardiovascular dysfunction. Our previous work showed that adipose‐derived extracellular vesicles (adiposomes) from obese donors impair arteriolar vasodilation through endothelial dysfunction, but their impact on vascular smooth muscle cell (VSMC) function remains unclear.

**Methods:**

Visceral adipose tissues were collected from 25 obese and 12 lean subjects undergoing bariatric and elective surgeries, and from high‐fat diet‐induced obesity (DIO) mice (*n* = 40). Adiposomes were isolated by ultracentrifugation, and arteriolar myogenic tone was assessed using pressure myography. Intracellular Ca^2+^, membrane potential, and reactive oxygen species (ROS) were measured in VSMCs.

**Results:**

Obese arterioles exhibited greater myogenic tone than lean controls, a response also observed in healthy vessels exposed to obese adiposomes. Native VSMCs from obese subjects showed amplified acetylcholine‐induced Ca^2+^ waves, a response also observed in cultured VSMCs exposed to adiposomes from obese humans or DIO mice. Membrane potential analysis showed that obese adiposomes impaired KATP channel function, attenuating pinacidil‐induced hyperpolarization while enhancing glibenclamide‐mediated depolarization. Obese adiposomes also elevated ROS levels in VSMCs, which were reduced by extracellular ROS scavenging, normalizing K_ATP_ channel function and Ca^2+^‐influx, thereby ameliorating arterial hypercontractility in obese specimens. Furthermore, depleting ceramides in obese adiposomes diminished their ability to induce hypercontractility, highlighting ceramide as a key mediator of obesity‐induced vascular dysfunction.

**Conclusions:**

These findings underscore a pathogenic form of vascular–adipose crosstalk in obesity, where adiposome‐mediated signaling alters VSMC excitability and vascular tone. Targeting this inter‐organ communication axis may offer new strategies to reverse obesity‐related vascular complications.

## Graphical abstract

Ceramide‐rich adiposomes released from dysfunctional adipose tissue in obesity mediate adipose–vascular crosstalk. These vesicles promote ROS generation, disrupt KATP channel activity, and dysregulate Ca^2+^ signaling, leading to vascular smooth muscle hypercontractility and vascular dysfunction.

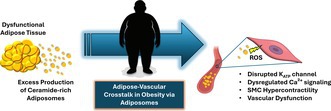

## 1 Introduction

Obesity, marked by abnormal adipose tissue expansion resulting from chronic energy surplus, has become a global pandemic, affecting over 650 million adults worldwide (Okunogbe et al. 2022). This condition represents a major risk factor for hypertension and cardiovascular diseases (CVDs), including coronary artery disease and atherosclerosis (Koenen et al. 2021; Powell‐Wiley et al. 2021; Hagberg and Spalding 2024). Beyond its metabolic consequences, such as insulin resistance and dyslipidemia, obesity profoundly disrupts vascular homeostasis, impairing vascular cell function (Hagberg and Spalding 2024; Valenzuela et al. 2023; Zhu et al. 2023; Ottolini et al. 2020).

Adipose tissue functions as an endocrine organ that secretes a wide range of mediators, either in soluble form or packed inside vesicles. In obesity, the altered metabolic activity of adipose tissue changes its secretome, contributing to the dysregulation of systemic and vascular physiology (Koenen et al. 2021; Powell‐Wiley et al. 2021; Hagberg and Spalding 2024; Landsberg et al. 2013; Van Gaal et al. 2006; Chait and den Hartigh 2020; Sakers et al. 2022). Adiposomes are increasingly recognized as crucial mediators in intercellular communication, transporting dysregulated cargo that contributes to vascular pathology (Hussein et al. 2024; Blandin et al. 2023; Le Lay et al. 2021). Unlike lipid droplets, intracellular organelles specialized for neutral lipid storage, adiposomes are extracellular vesicles secreted by adipose tissue that transport bioactive proteins, lipids, and nucleic acids, thereby serving as mediators of intercellular and interorgan communication (Blandin et al. 2023; Le Lay et al. 2021; Mahmoud et al. 2025). Our previous studies showed that adiposomes isolated from obese individuals induce endothelial dysfunction by compromising caveolae integrity and disrupting nitric oxide (NO) signaling in endothelial cells (Mirza et al. 2023; Mirza, Hassan, et al. 2022). These effects resulted in impaired vasodilation in arterioles treated with obese adiposomes. Others have shown that adiposomes from obese mouse models induce a phenotypic switch in vascular smooth muscle cells (VSMCs) from a contractile to a proliferating, pro‐inflammatory state (Li et al. 2019). However, the impact of obesity‐associated adiposomes on VSMCs contractility and Ca^2+^ signaling is yet to be explored.

VSMCs are essential regulators of vascular tone and blood pressure (Davis et al. 2023), and their dysfunction is a hallmark of obesity‐related CVDs (Shi et al. 2020). A key regulator of VSMC membrane potential and calcium influx is ATP‐sensitive K^+^ (K_ATP_) channels, an inwardly rectifying K^+^ channel sensitive to ATP levels. Active K_ATP_ hyperpolarizes the membrane and reduces Ca^2+^ entry. Conversely, inhibited K_ATP_ leads to membrane depolarization and increased Ca^2+^ influx, fueling VSMC contraction (Teramoto 2006). Dysregulated K_ATP_ has been implicated in hypertension (Dart 2014; Aziz et al. 2014; Nichols et al. 2013); however, the underlying cause of its dysregulation in obesity remains unclear. In obesity, adipose tissue inflammation and impaired glucose homeostasis produce abundant lipids, including ceramides, packaged into adiposomes (Hussein et al. 2024; Blandin et al. 2023; Mahmoud et al. 2025). Ceramides, a class of sphingolipids, are not only biomarkers of metabolic dysregulation but also active drivers of inflammation, oxidative stress, and cellular dysfunction (Cogolludo et al. 2019; Holland et al. 2011). Our recent work has demonstrated that obesity‐associated adiposomes carry more ceramides than lean adiposomes, which may directly impair vascular cell function (Hussein et al. 2024; Mahmoud et al. 2025; Mirza et al. 2023).

Unable to handle the excess lipids typical of obesity, VSMCs may experience disrupted contractile function following adiposome uptake. The impact of adiposomes and their dysregulated lipid content on vascular function remains largely unexplored despite adipose tissue being a primary source of lipids and metabolites that can profoundly influence other cells and tissues. This study aims to clarify how obesity‐associated adiposomes affect K_ATP_ channels in VSMCs, contributing to vascular hypercontractility, using an integrated approach that combines ex vivo analyses of arteriolar pressure myography and vascular assessments in preclinical and in vitro models. By revealing how obesity alters adiposomal signaling to disrupt VSMC homeostasis, we aim to fill a critical gap in understanding obesity‐induced vascular dysfunction. Examining vasoreactivity in small arterioles, key regulators of peripheral resistance and blood pressure, enhances the clinical relevance of this work. Together, this comprehensive approach has provided vital insights into how dysfunctional adipose tissue contributes to vascular risk in obesity.

## 2 Materials and Methods

### 2.1 Study Ethics Approval and Participants

The study was conducted following the Declaration of Helsinki and received approval from the Institutional Review Board of the University of Illinois Chicago (Protocol no. 2021–1113, approved October 2021). All participants provided written informed consent prior to enrollment in the study. Visceral adipose tissue biopsies were collected from 25 obese individuals undergoing bariatric surgeries (BMI > 30 kg/m^2^) and 12 non‐obese controls (BMI < 25 kg/m^2^) during elective surgeries at the University of Illinois Chicago Hospital. Participant characteristics are detailed in Table S1. Individuals were excluded if they reported current tobacco use, pregnancy, a prior history of metabolic surgery, or chronic medical conditions such as hepatic, renal, or cardiac disease, cancer, or acute/chronic inflammatory disorders.

### 2.2 Animal Model for Obesity

Adult male and female C57BL/6 mice (2–3 months old; RRID:IMSR_JAX:000664) were used. A total of 40 animals (20 per group, sex balanced) were assigned to standard diet (SD) or high‐fat diet‐induced obesity (DIO) groups. Power analyses (G*Power, α = 0.05, > 80% power) determined sample size requirements. Animals were housed under controlled temperature (22°C ± 1°C), humidity (50% ± 10%), and light cycles (12 h light/dark), with ≤ 5 mice per cage. The DIO group received a 60% kcal fat diet (Research Diets, no. D12492) ad libitum for 8–10 weeks. Body weight and food consumption were measured weekly. At study completion, male DIO mice typically weighed 45–50 g and females 35–45 g, compared with 20–25 g in SD controls. All procedures were approved by the Institutional Animal Care and Use Committee of the University of Illinois Chicago (Protocol no. 21–145) and adhered to ARRIVE guidelines. Mice showing illness, abnormal behavior, or failure to gain weight on the DIO were excluded.

### 2.3 Isolation and Characterization of Adiposomes

Fresh visceral adipose tissue biopsies were surgically collected from human participants and DIO and SD mice, following established protocols (Mirza et al. 2023; Han et al. 2024). Tissue fragments (7–10 mg, ~1–2 mm^3^) were enzymatically digested with 1 mg/mL collagenase type I (Worthington) in M199 medium containing 4% BSA, followed by incubation in a 37°C water bath for 60 min with agitation. The digested suspension was filtered, centrifuged (1000 × *g*, 1 min), and floating adipocytes were collected. Adipocytes were cultured in M199 medium supplemented with 5% exosome‐free fetal bovine serum and 1% penicillin/streptomycin for 48–72 h. In some experiments, cultures were treated with myriocin (serine palmitoyltransferase inhibitor; Millipore Sigma, CAS 35891‐70‐4) or GW4869 (neutral sphingomyelinase inhibitor; Millipore Sigma, CAS 6823‐69‐4) to reduce ceramide content. Conditioned media were centrifuged at 15,000 × *g* for 15 min, filtered (0.45 μm), and ultracentrifuged (150,000 × *g*, 120 min). Adiposome pellets were resuspended in PBS and quantified by nanoparticle tracking analysis (NTA) (NanoSight NS300, Malvern).

### 2.4 Transmission Electron Microscopy (TEM)

Adiposomes were fixed in 2% paraformaldehyde for 5 min, applied to Formvar/carbon‐coated copper grids, and immediately stained with 1% uranyl acetate. Samples were imaged using a JEM‐3010 transmission electron microscope.

### 2.5 Adiposome Uptake

Isolated adiposomes were labeled with BODIPY TR‐ceramide (Life Technologies; 589/617 nm excitation/emission) (Mirza et al. 2023). Briefly, a 1 mmol/L BODIPY stock solution was prepared, and 1 μL was added to every 100 μL of adiposome suspension, yielding a final concentration of 10 μmol/L. Following a 20 min incubation at 37°C, unbound dye was removed using Exosome Spin Columns (Life Technologies, MW 3000). Cultured VSMCs or intact vessels were incubated with labeled adiposomes for 30 min before fixation and staining. Cytoskeletal structures were visualized with Alexa Fluor 488 Phalloidin (Cell Signaling Technologies, no. 8878S), nuclei with DAPI, and VSMCs within vessels with anti‐α‐smooth muscle actin (α‐SMA) primary antibody (Abcam, ab5694, 1:100) followed by Alexa Fluor 488 conjugated goat anti‐rabbit secondary antibody (Thermo Fisher, A‐11008, 1:500). The whole‐mounted vessels were then placed on glass slides with DAPI mounting medium (Vector Laboratories, UX‐93952‐24). Images were acquired using a Leica Dmi8 fluorescence microscope equipped with a DFC3000 G camera and an 89 North PhotoFluor LM‐75 illumination system.

### 2.6 Isolation of Native VSMCs and Culture of Primary VSMCs

VSMCs were isolated from adipose arterioles of lean and obese subjects, as described previously (Metwally et al. 2024). Arterioles were dissected from adipose tissue biopsies and rinsed in Mg‐PSS. Native VSMCs were obtained by enzymatic digestion of the isolated vessels with 1 mg/mL papain (Worthington Biochemical Corporation), 1 mg/mL dithiothreitol (DTT), and 10 mg/mL BSA in Mg‐PSS at 37°C for 10 min, followed by a second 10 min digestion with 1 mg/mL collagenase type II (Worthington Biochemical Corporation). The resulting tissue was gently washed three times and triturated with a fire‐polished glass pipette to generate a single‐cell suspension. Cells were freshly dissociated on the day of the experiment and plated onto glass‐bottom dishes, where they were allowed to adhere for 30 min before use.

Human aortic smooth muscle cells (HAoSMCs) were cultured following manufacturer protocols (PromoCell, Heidelberg, Germany). Cryopreserved HAoSMCs (PromoCell, Cat. No. C‐12533) were thawed rapidly at 37°C and transferred to 9 mL of pre‐warmed Smooth Muscle Cell Growth Basal Medium (SmBM, Lonza, no. CC‐3181) supplemented with the SmGM‐2 BulletKit (Lonza, no. CC‐4149). Supplements included 5% fetal bovine serum (FBS), 0.5 mL insulin, 1.0 mL human fibroblast growth factor (hFGF‐B), 0.5 mL GA‐1000, and 0.5 mL human epidermal growth factor (hEGF). Cells were centrifuged at 300 × *g* for 5 min, resuspended in 1–2 mL complete medium, and seeded in gelatin‐coated (0.1%) T‐75 flasks. Cultures were maintained at 37°C in 5% CO_2_. At 70%–80% confluence, cells were passaged using a non‐enzymatic dissociation reagent (CellStripper, Corning, no. 25–056‐CI) to preserve surface protein integrity. Detached cells were centrifuged, resuspended in fresh medium, and seeded into 35 mm glass‐bottom dishes at a 1:2 to 1:3 ratio. For cryopreservation, cells were suspended in freezing medium (90% FBS + 10% DMSO), aliquoted into cryovials, frozen at −80°C overnight, and then transferred to liquid nitrogen for long‐term storage.

### 2.7 Pressure Myography

Pressure myography was performed in accordance with established best‐practice guidelines (Metwally et al. 2024; Wenceslau et al. 2021). Human adipose tissue biopsies and mouse mesenteric or skeletal muscle samples were placed in a Sylgard‐lined Petri dish containing Ca^2+^‐free Mg‐PSS supplemented with 0.5% BSA. Adipose resistance arterioles, third‐order mesenteric arteries, and small skeletal muscle arterioles were carefully dissected and cannulated onto glass micropipettes (~50–100 μm outer diameter) in a pressure myograph chamber (Living Systems Instrumentation), then secured with nylon thread. Vessel imaging was performed using an inverted microscope (Olympus CKX53) coupled to a CMOS USB camera (VESCAM2, 1440 × 1080, 1.6 MP, USB3.1), and luminal diameter was tracked with IonWizard software (version 7.2.7.138; IonOptix LLC, Westwood, MA, USA).

Arterioles were maintained at 37°C in continuously oxygenated PSS (21% O_2_, 6% CO_2_, 73% N_2_) with the following composition: 119 mM NaCl, 4.7 mM KCl, 21 mM NaHCO_3_, 1.17 mM MgSO_4_, 1.8 mM CaCl_2_, 1.18 mM KH_2_PO_4_, 5 mM glucose, and 0.03 mM EDTA. After equilibration at 5 mmHg for 15 min, vessels were pressurized to 110 mmHg, stretched to approximate in vivo length, and returned to 5 mmHg for an additional 15 min. Viability was assessed by constriction to high extracellular [K^+^] PSS (60 mM KCl, 63.7 mM NaCl). Arteries failing to constrict by at least 10% were excluded from analysis.

Human adipose arterioles were incubated with adiposomes derived from lean or obese donors, while mouse mesenteric arterioles were treated with adiposomes from lean or diet‐induced obese (DIO) mice. All incubations were performed for 5 h at 37°C. In separate experiments, mesenteric vessels were exposed to DIO‐derived adiposomes alone or together with the nitric oxide (NO) donor sodium nitroprusside (SNP, 10 μM). Endothelial integrity was assessed by gently perfusing air through the lumen to induce denudation.

Myogenic tone was assessed by stepwise increases in intraluminal pressure: 5–20 mmHg, then in 20 mmHg increments up to 140 mmHg. At each level, vessels were allowed to develop spontaneous constriction until a stable diameter was reached. Active diameters were recorded, after which pressure was reduced to 5 mmHg. Passive diameters were determined by superfusing vessels with Ca^2+^‐free PSS containing EGTA (2 mM) and diltiazem (10 μM). Myogenic tone (%) was calculated as: Myogenic tone (%) was calculated as [1 − (active diameter/passive diameter)] × 100. The distensibility coefficient, representing the ability of the vessel wall to expand with pressure, was calculated as: =2*(maximum diameter‐ baseline diameter)/baseline diameter/(maximum pressure‐minimum pressure) (Metwally et al. 2024).

### 2.8 Ca^2+^ Imaging and Analysis

Intracellular Ca^2+^ dynamics were evaluated in VSMCs freshly isolated from lean and obese individuals, as well as in cultured VSMCs exposed to adiposomes from lean or obese donors. The fluorescent Ca^2+^ indicator Fluo‐4 AM (5 μM; Invitrogen, Carlsbad, CA, no. F14201) was used to load cells for 30 min at 37°C. Following dye loading, cells were washed and transferred into a Ca^2+^‐free buffer. Imaging was performed on a ZEISS LSM 710 confocal microscope with excitation at 488 nm, using a 40× water‐immersion objective. Images were acquired at 2 frames/s. In specific experiments, cells were pre‐incubated with thapsigargin (TG, 1 μM; Sigma‐Aldrich, St. Louis, MO) for 15 min prior to imaging. To stimulate Ca^2+^ responses in freshly isolated VSMCs, acetylcholine (ACh, 5 μM) was applied.

Ca^2+^ recordings were analyzed with SparkAn software (developed by Dr. Adrian Bonev, University of Vermont) (Dabertrand et al. 2012). Baseline fluorescence (F₀) was defined as the mean of the first 10 frames prior to stimulation. Localized increases in fluorescence (ΔF) within defined regions of interest (ROI, 3 × 3 μm^2^) were identified as discrete Ca^2+^ events, characterized by transient peaks in intensity over time. ΔF/F₀ traces were generated for multiple sites within each recording, and the following parameters were quantified: peak amplitude (ΔF/F₀), frequency (Hz), and event duration (Metwally et al. 2024).

### 2.9 Membrane Potential Assay

Changes in membrane potential were measured using the fluorescent probe Bis‐(1,3‐dibutylbarbituric acid) trimethine oxonol (DiBAC_4_ (3); Invitrogen, Cat. No. B438). Cultured VSMCs were loaded with 10 μM DiBAC_4_ (3) for 30 min at 37°C. Fluorescence was first recorded under baseline conditions and then after exposure to the K_ATP_ channel opener pinacidil (10 μM; Cayman Chemical) or the channel blocker glibenclamide (10 μM; Tocris Bioscience).

### 2.10 Reactive Oxygen Species (ROS) Measurement

ROS production was evaluated using the fluorescent probe CM‐H_2_DCFDA (10 μM, Invitrogen, Cat. No. C6827) (Metwally et al. 2024). Cultured VSMCs were exposed to adiposomes isolated from either lean or obese donors and subsequently incubated with CM‐H_2_DCFDA for 30 min at 37°C. For mechanistic studies, cells were pretreated for 15 min at 37°C with specific ROS scavengers, including superoxide dismutase (SOD; Sigma‐Aldrich, S9697‐30KU), catalase (Sigma‐Aldrich, C40), or polyethylene glycol–conjugated SOD (PEG‐SOD; Sigma‐Aldrich, S9549). These agents were maintained in the medium throughout the experiment to preserve their activity while cells were continuously challenged with adiposomes. Fluorescence imaging was performed on a ZEISS LSM 710 confocal microscope with excitation at 488 nm, using a 40× water‐immersion objective. Images were acquired at 2 frames/s. Signal intensities were analyzed in ImageJ (NIH, Bethesda, MD, USA; http://www.imagej.nih.gov/ij/, RRID:SCR_003070). Fluorescence traces were normalized to baseline values (ΔF/F₀), and time‐dependent changes in ROS generation were quantified for each recording.

### 2.11 Chemicals

Unless otherwise specified, all reagents and chemicals were purchased from Sigma‐Aldrich Inc.

### 2.12 Statistical Analysis

All statistical analyses were conducted with GraphPad Prism v9.4.1. Data are expressed as mean ± SEM. The sample size (“*n*”) indicates the number of independent biological replicates, where each replicate represents averaged values from vessels or cells obtained from a single donor or mouse. Statistical comparisons included the Student's *t* test, one‐way analysis of variance (ANOVA) with Tukey's multiple comparisons test, and two‐way ANOVA with Šídák's multiple comparisons test. Significance was defined as *p < 0.05, **p < 0.01, and ***p < 0.001.

### 2.13 Data and Materials Availability

The manuscript and supporting informations provide all the necessary data to assess the conclusions of the study. Original datasets, together with individual comparison analyses, are provided in the supporting data values file.

## 3 Results

### 3.1 Small Adipose Arterioles From Obese Individuals Exhibit Hypercontractility

**FIGURE 1:**
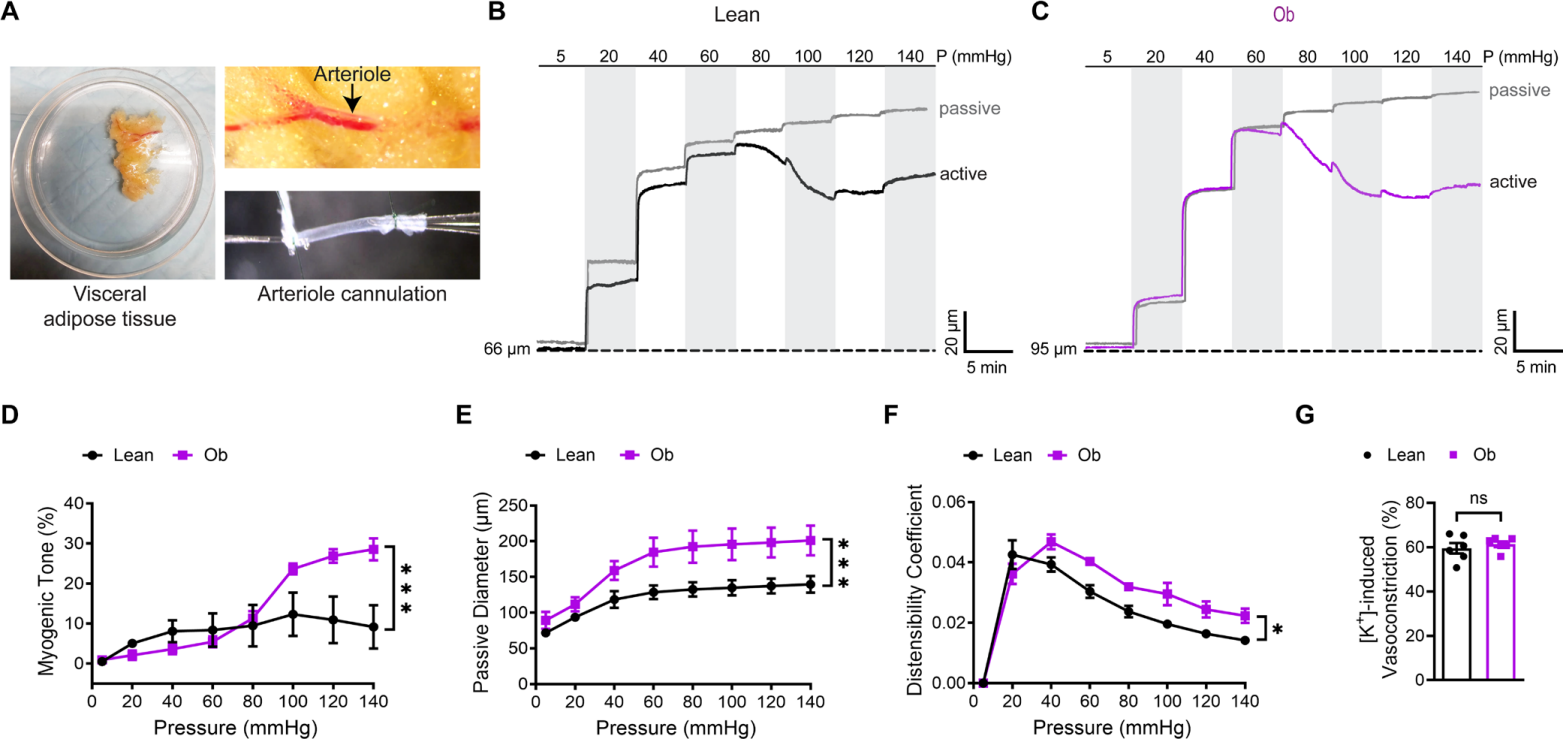
Small adipose arterioles from obese individuals exhibit hypercontractility. (A) Isolation of a small arteriole (arrow) from human visceral adipose tissue. The vessel is gently cannulated onto glass micropipettes and secured with nylon sutures. (B, C) Representative intraluminal diameter tracings of adipose arterioles from lean and obese (Ob) individuals in response to stepwise intraluminal pressure increases (5–140 mmHg), recorded under active (Ca^2+^‐containing solution) and passive (Ca^2+^‐free solution) conditions. (D) Summary of myogenic tone (%). Data are mean ± SEM; *n* = 6 vessels (2 vessels from each of 3 individuals per group). ***p < 0.001, two‐way ANOVA with Šidák's multiple comparisons test. (E, F) Passive inner diameter (μm) and distensibility coefficient of isolated arterioles measured under Ca^2+^‐free conditions (2 mM EGTA +10 μM diltiazem). Data are mean ± SEM; *n* = 6 vessels per group. *p < 0.05, ***p < 0.001, two‐way ANOVA with Šidák's multiple comparisons test. (G) Constriction responses of isolated arterioles to 60 mM KCl. Data are mean ± SEM; *n* = 6 vessels per group. ns, not significant (*p* ≥ 0.05, unpaired Student's *t* test).

To investigate the contractile properties of small adipose arterioles isolated from obese individuals, the myogenic tone was evaluated by pressure myography (Wenceslau et al. 2021), where VSMC contraction was determined from luminal diameter changes during stepwise intraluminal pressure increases (5–140 mmHg) in Ca^2+^‐containing solution, followed by Ca^2+^‐free perfusion to obtain passive diameters, with tone calculated as the normalized difference between active and passive values (Figure 1A). Arterioles isolated from obese individuals demonstrated significantly elevated myogenic tone compared to those from lean controls (*p* < 0.001; Figure 1B–D). This hypercontractility was first detectable at 100 mmHg (24% ± 1.8% for obese vs. 12% ± 3.6% for lean, *p* = 0.014; Figure 1D) and became progressively more pronounced at higher pressures, reaching maximal differences at 140 mmHg (29.6% ± 3.3% for obese vs. 9% ± 3% for lean, *p* < 0.001; Figure 1D). In Ca^2+^‐free conditions, arterioles from obese individuals exhibited a significantly larger passive diameter than lean controls (*p* < 0.001; Figure 1E). Consistent with this mechanical alteration, the distensibility coefficient, reflecting the vessel's ability to expand under pressure, was significantly elevated in obese individuals compared with controls (*p* = 0.024; Figure 1F).

To determine whether voltage‐gated calcium channels (VGCCs) contribute to increased vascular tone in obesity, we compared KCl (60 mM)‐induced vasoconstriction, a depolarization‐dependent VGCC activation, in arterioles from lean and obese subjects. Contractile responses were similar between groups (*p* = 0.609; Figure 1G), demonstrating that obesity does not alter intrinsic VGCC‐mediated constriction under these experimental conditions. These findings indicate that the elevated myogenic tone observed in obesity is not attributable to direct alterations in VGCC function. Instead, the findings suggest that obesity‐associated changes in upstream signaling pathways may underlie the enhanced vascular tone.

### 3.2 Obesity‐Associated Adiposomes Acutely Increase Arteriole Contractility

**FIGURE 2:**
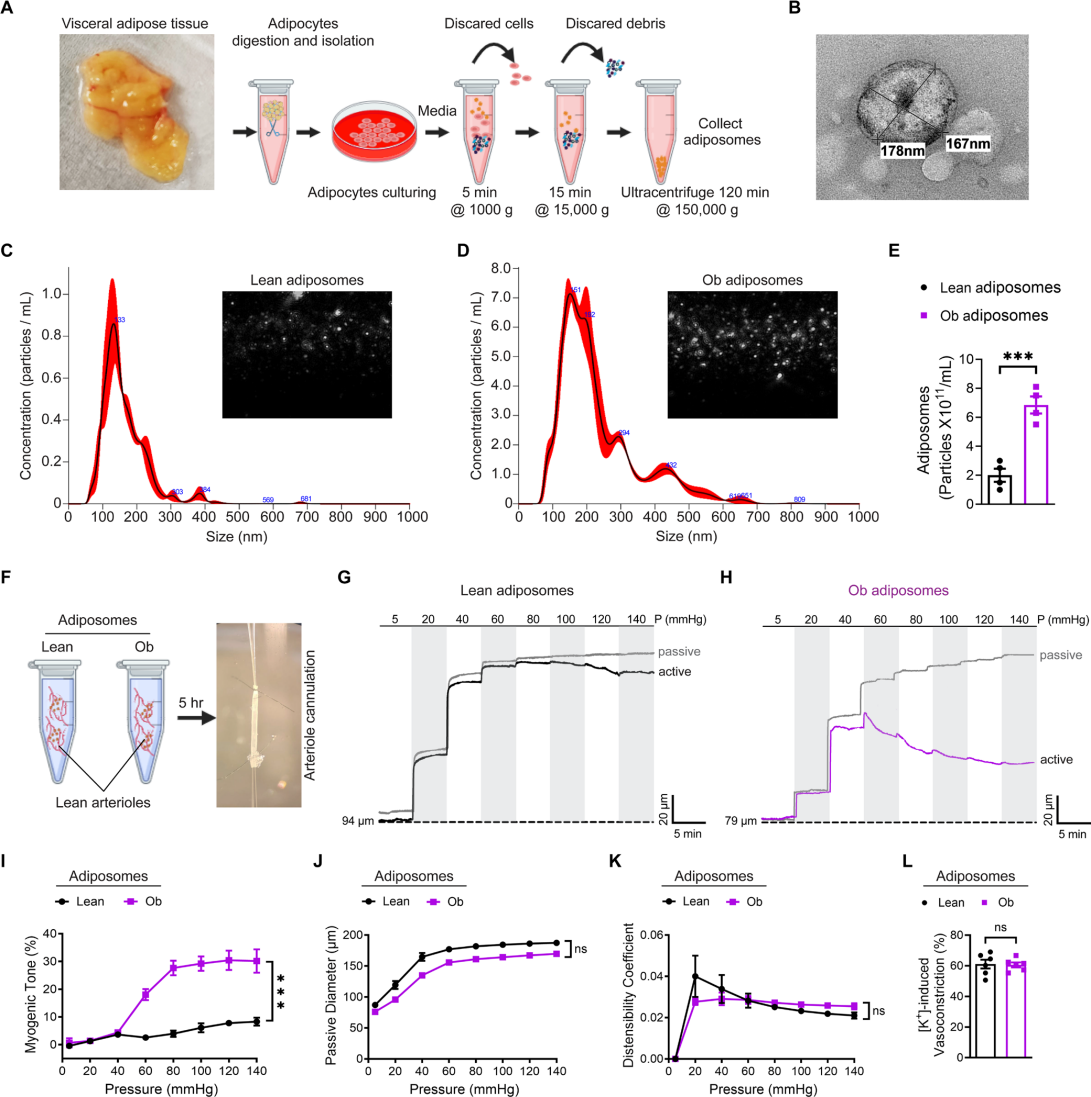
Obesity‐associated adiposomes acutely increase arteriole contractility. (A) Schematic of adiposome isolation from visceral adipose tissue, including centrifugation and purification steps. (B) Representative TEM image showing isolated adiposomes indicated by their dimensions. (C, D) Adiposome production is increased in obese (Ob) individuals compared to lean controls. Representative NTA graphs and images showing the size distribution and concentration of adiposomes isolated from lean and obese individuals, analyzed using the NanoSight NS300 system. (E) Quantification of adiposome particle number. Data are mean ± SEM; *n* = 4 independent experiments. ***p < 0.001, unpaired Student's *t* test. (F) Schematic showing isolated adipose arterioles from healthy controls incubated with adiposomes from lean or obese individuals for 5 h at 37°C before cannulation into glass micropipettes. (G, H) Representative tracings of intraluminal diameter changes in healthy arterioles after co‐incubation with adiposomes from lean or obese donors. (I) Myogenic tone (%) summary. Data are mean ± SEM; *n* = 6 vessels (2 vessels from each of 3 individuals per group). ***p < 0.001, two‐way ANOVA with Šidák's multiple comparisons test. (J, K) Passive inner diameter (μm) and distensibility coefficient of arterioles. Data are mean ± SEM; *n* = 6 vessels from 3 individuals per group. Two‐way ANOVA with Šidák's test; ns, not significant (*p* ≥ 0.05). (L) Constriction of isolated arterioles in response to 60 mM KCl. Data are mean ± SEM; *n* = 6 arteries. Unpaired Student's *t* test; ns, not significant (*p* ≥ 0.05).

To investigate the potential influence of obesity‐associated adiposomes on small arteriole contractility, adiposomes were isolated from lean and obese adipose tissue through a series of centrifugation and purification steps (Figure 2A). Transmission electron microscopy confirmed the successful isolation of intact adiposomes, revealing the cup‐shaped morphology typical of extracellular vesicles (Figure 2B). For further characterization, NTA was performed using the NanoSight NS300 system (Mirza et al. 2023). This analysis quantified adiposome size distribution and concentration, demonstrating a significantly higher particle concentration in adiposomes derived from obese individuals than lean subjects (Figure 2C–E).

Arterioles isolated from lean individuals were incubated with adiposomes derived from lean or obese donors, and changes in luminal diameter were assessed under active and passive conditions (Figure 2F). Arterioles treated with obese adiposomes exhibited a significant increase in contractility compared to those exposed to lean adiposomes (*p* < 0.001; Figure 2G–I). The increased contractility first became statistically significant at 60 mmHg and progressively increased with rising pressure (19.4% ± 2% for obese adiposomes vs. 1.9% ± 0.6% for lean adiposomes, *p* < 0.001; Figure 2I). Passive diameter and distensibility measurements in arterioles acutely exposed to obese adiposomes showed no significant differences relative to those treated with lean adiposomes (*p* = 0.859, *p* = 0.785; Figure 2J,K). These findings suggest there was no observable compensatory adjustment in vascular mechanics under the conditions of acute adiposome treatment.

To assess whether isolated adiposomes influence the intrinsic activity of VGCCs, vasoconstriction was induced by direct membrane depolarization with 60 mM KCl. Arterioles treated with adiposomes from lean or obese donors exhibited comparable KCl‐induced constriction (*p* = 0.093; Figure 2L), suggesting that adiposome treatment does not affect intrinsic VGCC function.

### 3.3 DIO Adiposomes Induce Hypercontractility in Small Peripheral Arterioles of Mice

**FIGURE 3:**
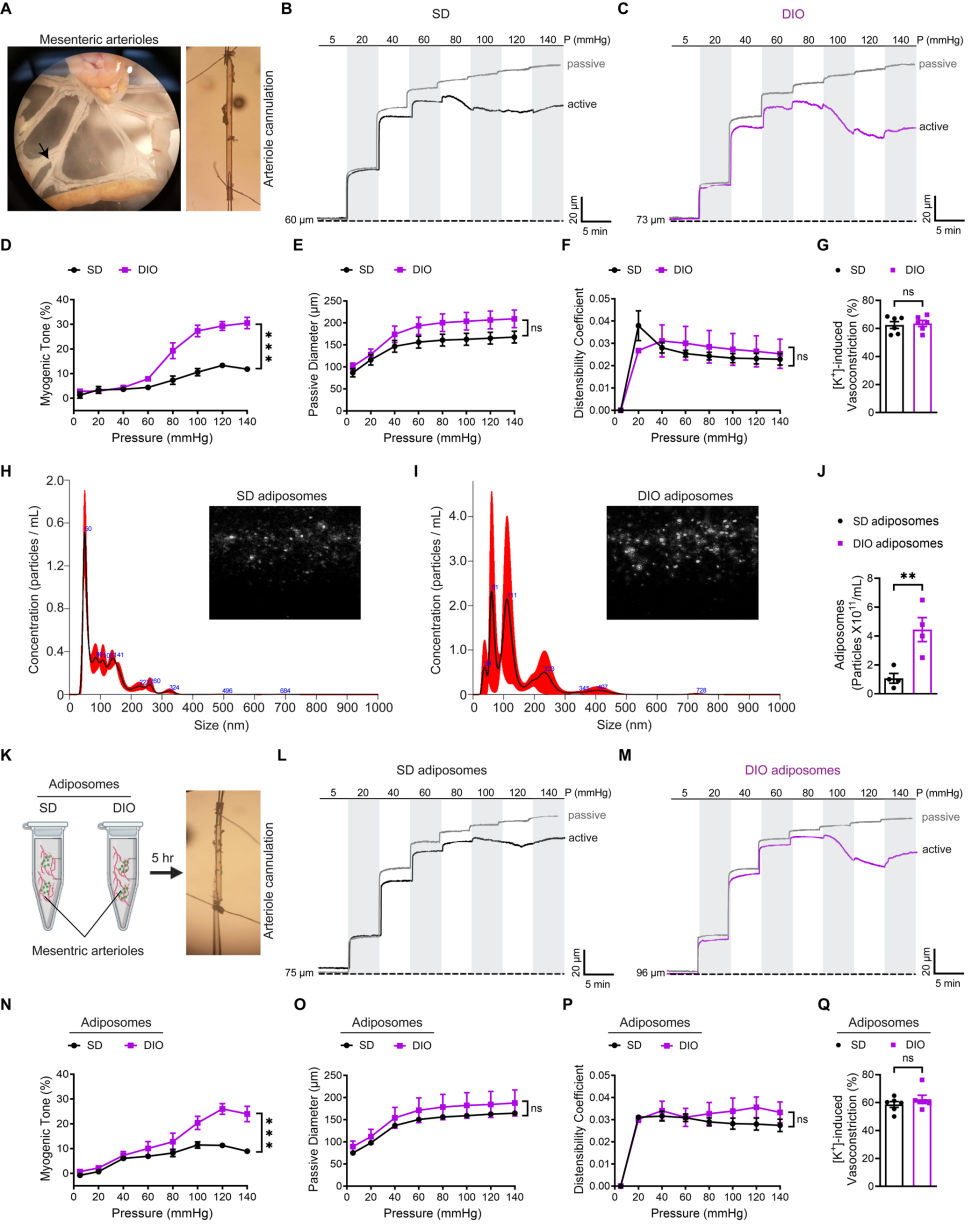
DIO‐associated adiposomes induce hypercontractility in peripheral small arterioles of mice. (A) Isolation of a 3rd‐order mesenteric arteriole (arrow) from mice for pressure myography. (B, C) Representative tracings of intraluminal diameter changes in mesenteric arterioles from SD and DIO mice. (D) Myogenic tone (%) summary data. Values are mean ± SEM; *n* = 6 vessels (2 per mouse, 3 mice/group). ***p < 0.001, two‐way ANOVA with Šidák's multiple comparisons test. (E, F) Passive inner diameter (μm) and distensibility coefficient of mesenteric arterioles. Data are mean ± SEM; *n* = 6 vessels from 3 mice/group. ns, not significant (*p* ≥ 0.05), two‐way ANOVA with Šidák's test. (G) Vasoconstriction to 60 mM KCl. Data are mean ± SEM; *n* = 6 arteries. ns, not significant (*p* ≥ 0.05), unpaired Student's *t* test. (H, I) Increased adiposome production in DIO mice compared with SD controls, shown by NTA graphs and images of size distribution and concentration. (J) Quantification of adiposome particle numbers. Data are mean ± SEM; *n* = 4 independent experiments. **p < 0.01, unpaired Student's *t* test. (K) Schematic of isolated 3rd‐order mesenteric arterioles from controls incubated with adiposomes from SD or DIO mice for 5 h. (L, M) Representative tracings of diameter changes in mesenteric arterioles co‐incubated with SD‐ or DIO‐derived adiposomes. (N) Myogenic tone (%) summary data. Values are mean ± SEM; *n* = 6 vessels (2 per mouse, 3 mice/group). ***p < 0.001, two‐way ANOVA with Šidák's multiple comparisons test. (O, P) Passive inner diameter (μm) and distensibility coefficient. Data are mean ± SEM; *n* = 6 vessels from 3 mice/group. ns, not significant (*p* ≥ 0.05), two‐way ANOVA with Šidák's test. (Q) Constriction of mesenteric arterioles to 60 mM KCl. Data are mean ± SEM; *n* = 6 arteries. ns, not significant (*p* ≥ 0.05), unpaired Student's *t* test.

To investigate the vascular consequences of obesity in a controlled experimental setting, we utilized the DIO mouse model, which allows for isolating obesity‐specific effects while minimizing confounding variables usually seen in clinical samples. Small mesenteric and skeletal muscle arterioles, key resistance vessels involved in blood flow regulation and peripheral vascular resistance (Wenceslau et al. 2021), were isolated and subjected to pressure myography. These vascular beds are particularly relevant for studying obesity‐related dysfunction due to their role in metabolic and hemodynamic regulation (Zhu et al. 2023; Ottolini et al. 2020). Mesenteric and skeletal muscle arterioles from DIO mice exhibited significantly enhanced myogenic tone compared to SD controls (Figure 3A–D; Figure S1). This hypercontractility became evident at higher pressures in both vascular beds (≥ 80 mmHg, *p* < 0.001 for mesenteric vessels; ≥ 100 mmHg, *p* = 0.04 for skeletal muscle arterioles; Figure 3D; Figure S1) and progressively intensified, suggesting a pressure‐dependent exacerbation of vascular dysfunction in obesity. To assess structural adaptations, passive diameter and distensibility were evaluated in Ca^2+^‐free conditions. Both vascular beds showed no significant differences when comparing DIO and SD groups (Figure 3E,F; Figure S1), indicating that the hypercontractility due to DIO was not associated with vessel stiffness. To rule out the impact of DIO on intrinsic VGCC function, arterioles were exposed to 60 mM KCl to induce depolarization‐dependent vasoconstriction. Contractile responses did not differ significantly between DIO and SD mice in either vascular bed, indicating preserved VGCC intrinsic function (Figure 3G; Figure S1).

Adiposomes were also isolated from visceral adipose tissue of DIO and SD mice to examine their impact on vascular contractility. Analysis revealed a significantly higher concentration of adiposome particles in DIO mice than in SD controls, further supporting the link between obesity and elevated adiposome production observed in the clinical samples (Figure 3H–J). Isolated adiposomes were incubated with mesenteric arterioles from lean, healthy littermates (Figure 3K). Arterioles treated with DIO‐derived adiposomes exhibited significantly increased contractility compared to those exposed to SD adiposomes (*p* < 0.001, *p* = 0.02 for 100 mmHg, *p* = 0.0001 for 140 mmHg; Figure 3L–N), without alterations in passive diameter or distensibility (*p* = 0.99, *p* = 0.75; Figure 3O,P). KCl‐induced constriction remained unchanged (*p* = 0.61; Figure 3Q), further supporting that adiposome‐induced hypercontractility is independent of VGCC dysfunction. These findings demonstrate that DIO leads to heightened arteriolar contractility, likely mediated by adiposome‐dependent mechanisms unrelated to structural remodeling or intrinsic VGCC alterations.

To further isolate the effect of adiposomes on VSMCs, we tested whether the NO donor SNP could partially or completely reverse adiposome‐induced hypercontractility. We found that the hypercontractile response induced by adiposomes isolated from DIO mice persisted despite SNP treatment (Figure S2A). Endothelial disruption by air perfusion did not eliminate the hypercontractile phenotype but further augmented it (Figure S2B). This observation was supported by evidence of adiposome uptake by VSMCs in intact vessels, as shown by the accumulation of BODIPY‐labeled adiposomes after 30 min of incubation (Figure S3). These findings suggest that adiposomes exert their effects directly on VSMCs, independent of endothelial influence.

### 3.4 Enhanced Ca^2+^ Influx in Native VSMCs From Obese Individuals

**FIGURE 4:**
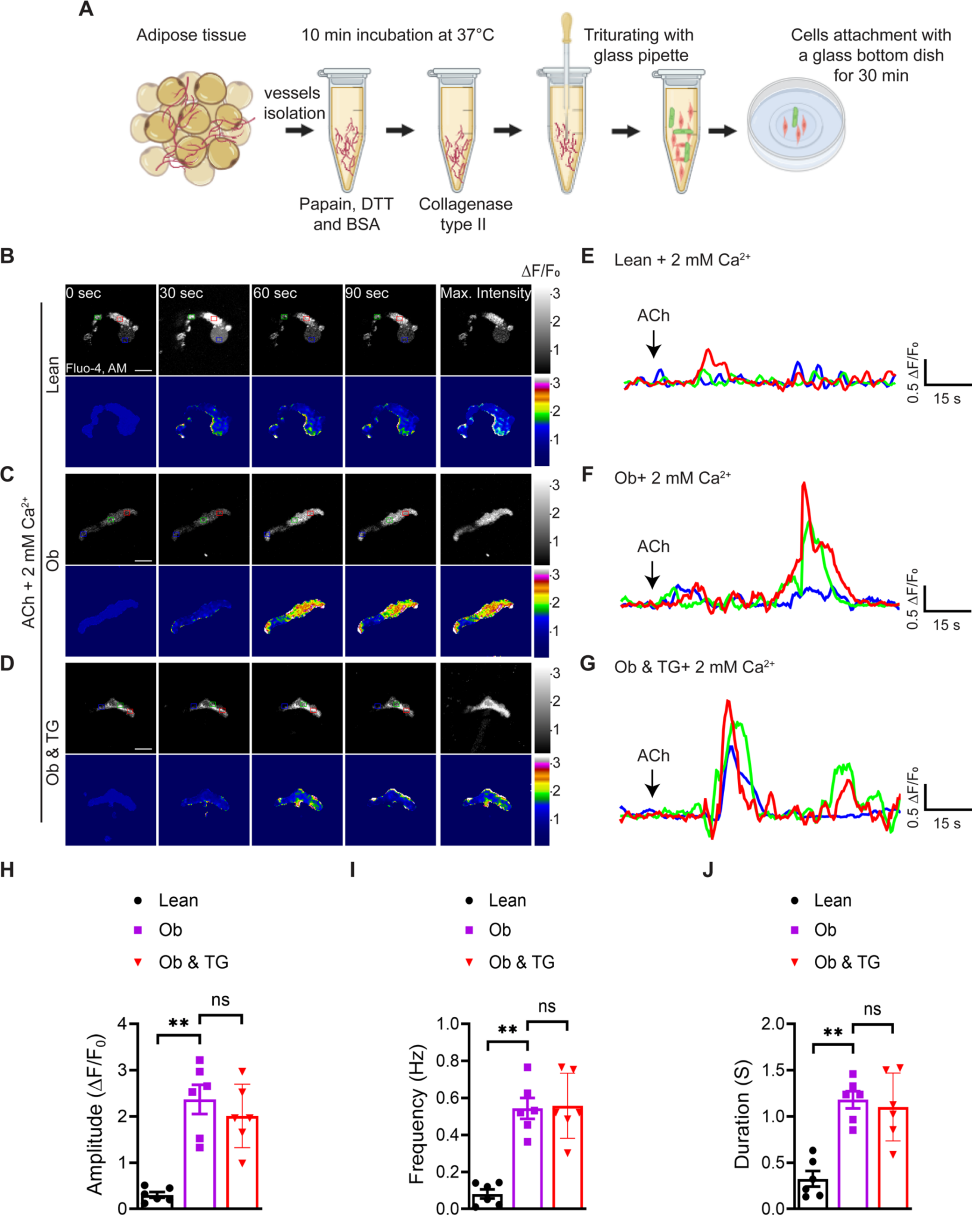
Enhanced Ca^2+^ influx in native VSMCs from obese individuals. (A) Schematic of native VSMC isolation from adipose arterioles by enzymatic digestion and mechanical trituration. (B–D) Representative grayscale and pseudocolored images of native VSMCs from lean and obese (Ob) individuals. Cells were loaded with Fluo‐4 AM, baseline fluorescence was recorded for 15 s, and then they were stimulated with ACh (5 μM) in Ca^2+^‐containing solution (B, C) or pretreated with thapsigargin (TG, 2 μM) (D) or pretreated with thapsigargin (TG, 2 μM) before ACh stimulation (E–G) or pretreated with thapsigargin (TG, 2 μM) before ACh stimulation (H–J) Summary data for Ca^2+^ signal amplitude (ΔF/F₀), frequency (Hz), and duration in VSMCs. Data are mean ± SEM; *n* = 6 cells (2 cells from each of 3 individuals per group). **p < 0.01, two‐way ANOVA with Tukey's multiple comparisons test; ns, not significant (*p* ≥ 0.05).

Calcium signaling is pivotal in regulating myogenic tone, with intracellular Ca^2+^ levels directly controlling vSMC contraction (Davis et al. 2023; Jackson 2021). To investigate obesity‐associated alterations in Ca^2+^ signaling, native VSMCs were isolated from adipose arterioles of obese and lean individuals using enzymatic‐mechanical digestion (Figure 4A), followed by live‐cell Ca^2+^ imaging with the fluorescent indicator Fluo‐4 AM. Compared to lean controls, VSMCs isolated from obese individuals displayed markedly enhanced ACh‐induced Ca^2+^ responses (Figure 4B,C,E,F; Videos S1 and S2), characterized by significantly greater signal amplitude (0.35 ± 0.02 vs. 2.3 ± 0.12, *p* = 0.002; Figure 4H), higher frequency (0.09 ± 0.01 vs. 2.3 ± 0.12, *p* = 0.002; Figure 4I), and prolonged event duration (0.42 ± 0.09 vs. 1.17 ± 0.19, *p* = 0.009; Figure 4J). These findings suggest that elevated intracellular Ca^2+^ signaling may contribute to the hypercontractile phenotype observed in obesity‐associated vascular dysfunction.

Because ACh stimulates Ca^2+^ release from the endoplasmic reticulum (ER) in VSMCs, we performed additional experiments to probe the underlying mechanisms in obese cells. To evaluate the role of ER Ca^2+^ stores, cells were pretreated with thapsigargin (TG), an inhibitor of the sarcoplasmic/endoplasmic reticulum Ca^2+^‐ATPase (SERCA) pump, to induce ER Ca^2+^ depletion. Notably, the elevated Ca^2+^ amplitude, frequency, and duration in obese VSMCs persisted despite ER Ca^2+^ depletion (*p* = 0.632, 0.984, and 0.893, respectively; Figure 4D,G,H,J; Video S3), suggesting that the enhanced Ca^2+^ signaling is not driven by ER Ca^2+^ release but is likely due to increased extracellular Ca^2+^ influx.

### 3.5 Obesity‐Associated Adiposomes Acutely Increase Ca^2+^ Influx in Cultured VSMCs

**FIGURE 5:**
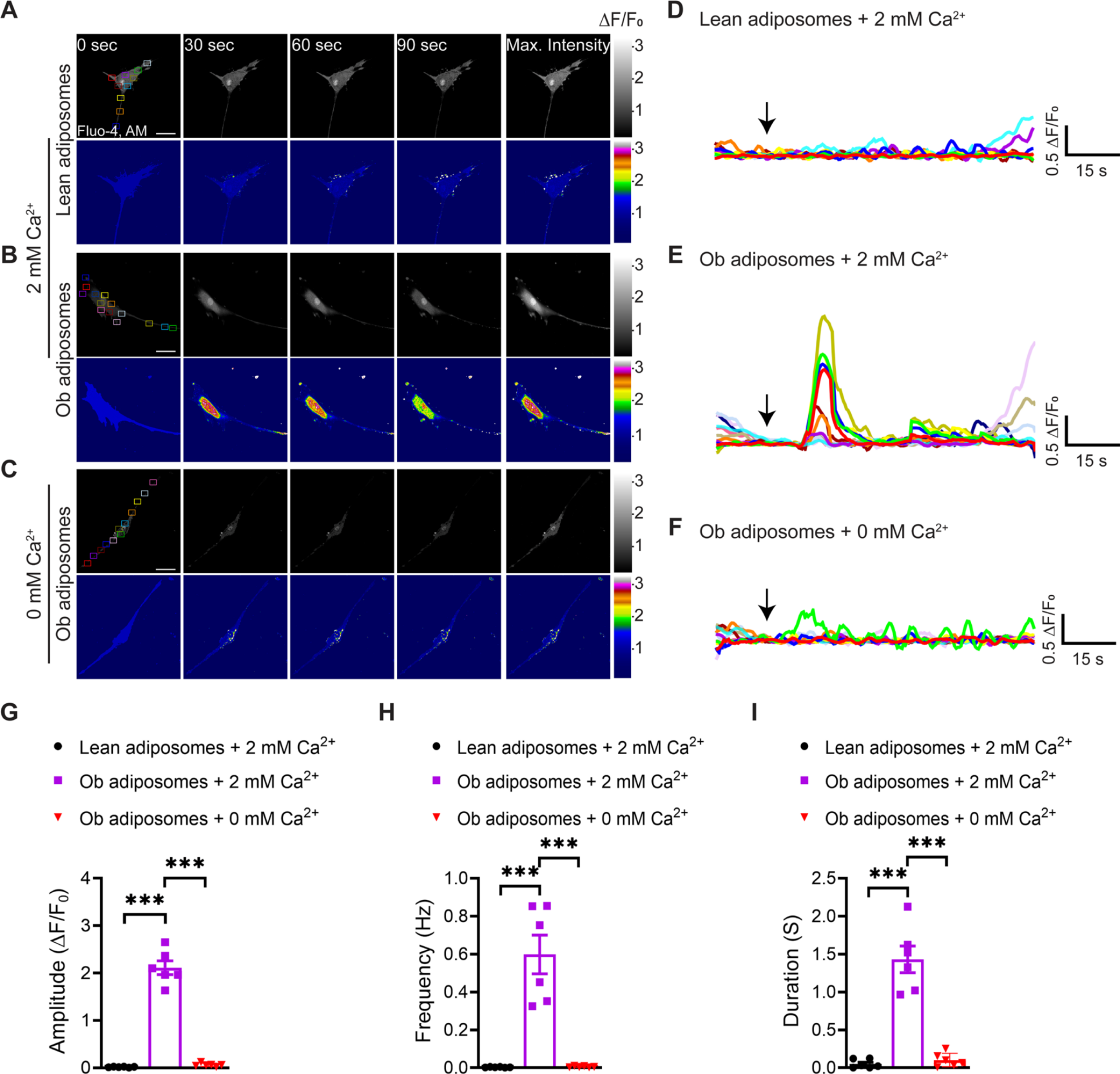
Obesity‐associated adiposomes acutely increase Ca^2+^ influx in cultured VSMCs. (A–C) Representative grayscale and pseudocolored images of cultured VSMCs treated with adiposomes from lean or obese (Ob) individuals. Cells were loaded with Fluo‐4 AM, baseline fluorescence was recorded for 15 s, and then cells were exposed to adiposomes for 90 s in Ca^2+^‐containing solution (A and B) or a Ca^2+^‐free solution (C). ROIs with active Ca^2+^ signals are highlighted by colored boxes. Scale bar = 50 μm. (D–F) Representative ΔF/F₀ vs. time plots showing Ca^2+^ traces from multiple ROIs under the indicated conditions. (G–I) Summary data for Ca^2+^ signal amplitude (ΔF/F₀), frequency (Hz), and duration in VSMCs. Data are mean ± SEM; *n* = 6 cells (imaged from independent preparations). ***p < 0.001, two‐way ANOVA with Tukey's multiple comparisons test.

Adiposome uptake was evaluated by incubating cultured VSMCs with the fluorescent dye BODIPY‐labeled adiposomes for 30 min. The uptake of adiposomes derived from obese individuals was significantly higher than that of lean individuals (Figure S4). To further investigate the impact of obesity‐associated adiposomes on Ca^2+^ signaling in VSMCs, cultured VSMCs were treated with adiposomes isolated from lean and obese individuals. Obese donor‐derived adiposomes triggered a marked increase in Ca^2+^ transient amplitude, frequency, and duration compared with lean donors (Figure 5A,B,D,E,G–I; Videos S4 and S5). Notably, this adiposome‐induced Ca^2+^ response was critically dependent on extracellular Ca^2+^, as the enhanced Ca^2+^ signaling was abolished entirely in VSMCs incubated in Ca^2+^‐free solution (Figure 5C,F,G–I; Video S6). Similarly, DIO adiposomes significantly increased the amplitude, frequency, and duration of Ca^2+^ signals compared to SD adiposomes (Figure S5; Videos S7 and S8). This effect was markedly dependent on extracellular Ca^2+^, as DIO adiposomes induced robust Ca^2+^ transients in the presence of TG but failed to trigger Ca^2+^ signals in Ca^2+^‐free solution (Figure S5; Videos S9 and S10). These results indicate that obesity‐associated adiposomes potently enhance Ca^2+^ influx in VSMCs, primarily through a mechanism involving extracellular Ca^2+^ entry rather than intracellular Ca^2+^ store release.

### 3.6 Obesity‐Associated Adiposomes Inhibit K_ATP_ Channel Activity in Cultured VSMCs

**FIGURE 6:**
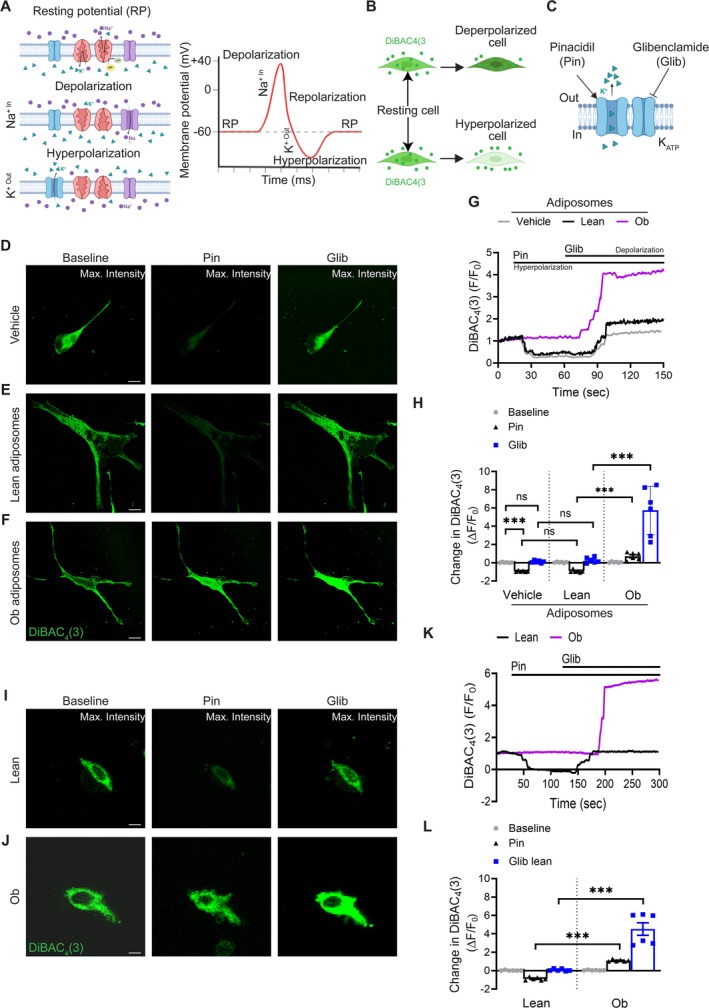
Obesity‐associated adiposomes inhibit K_ATP_ channel activity in cultured VSMCs. (A) The resting potential (RP) is maintained by balanced Na^+^ and K^+^ fluxes. Depolarization occurs via Na^+^ influx, whereas hyperpolarization results from K^+^ efflux. (B) Schematic of DiBAC_4_ (3) mechanism. The dye enters depolarized cells (positive potential), binds intracellularly, and increases fluorescence; in hyperpolarized cells (negative potential), it is excluded, reducing fluorescence. (C) Pharmacological modulation of K_ATP_ channels. Pinacidil (Pin, 10 μM) activates K_ATP_ channels, promoting K^+^ efflux and hyperpolarization; glibenclamide (Glib, 10 μM) blocks them, leading to depolarization. (D–F) Representative images of cultured VSMCs showing maximum DiBAC_4_ (3) fluorescence intensity under the indicated treatments (lean vs. obese adiposomes ± Pin or Glib). Scale bar = 50 μm.G, Representative plots of F/F₀ versus time for DiBAC_4_ (3) fluorescence under each indicated condition. (H) Quantification of changes (ΔF/F₀) in DiBAC_4_ (3) fluorescence intensity. Data are mean ± SEM; *n* = 6 cells per group (from independent preparations). ***p < 0.001, two‐way ANOVA with Tukey's multiple comparisons test; ns, not significant (*p* ≥ 0.05). (I, J) Representative images of isolated native VSMCs showing maximum DiBAC_4_ (3) fluorescence. Scale bar = 50 μm. (K) Representative traces of F/F₀ versus time for DiBAC_4_ (3) fluorescence. (L) Summary data of changes (ΔF/F₀) in DiBAC_4_ (3) fluorescence. Data are mean ± SEM; *n* = 6 cells (2 cells from each of 3 individuals per group). ***p < 0.001, two‐way ANOVA with Tukey's multiple comparisons test.

Given that obesity‐associated adiposomes enhance Ca^2+^ signaling in VSMCs primarily via increased Ca^2+^ influx and that vessels from obese individuals demonstrate heightened contractility, we next investigated the potential involvement of potassium (K^+^) channels in this dysregulated vascular response (Figure 6A). In particular, because membrane potential governs VSMC function by regulating Ca^2+^ entry and depolarization, both critical determinants of myogenic tone (Davis et al. 2023), we sought to examine the implication of K_ATP_ channels, a subset of K^+^ channels sensitive to metabolic alterations in adiposome‐induced Ca^2+^ influx and the consequent hypercontractility observed in obesity.

To test this hypothesis, we evaluated the effects of obesity‐associated adiposomes on K_ATP_ channel activity in cultured VSMCs by monitoring membrane potential dynamics using the fluorescent voltage‐sensitive dye DiBAC_4_ (3) (bis‐(1,3‐dibutylbarbituric acid)trimethine oxonol; Figure 6B). DiBAC_4_ (3) is a voltage‐sensitive dye that accumulates in depolarized cells, where it binds intracellular proteins and exhibits increased fluorescence, while hyperpolarization reduces its uptake and fluorescence intensity. Membrane potential was pharmacologically modulated using pinacidil, a K_ATP_ channel opener, and glibenclamide, a channel inhibitor, with corresponding changes in DiBAC_4_ (3) fluorescence intensity serving as a quantitative indicator of membrane potential dynamics (Figure 6C).

As expected, in untreated control conditions, pinacidil significantly reduced DiBAC_4_ (3) fluorescence compared to baseline (−0.93 ± 0.12 vs. 0.045 ± 0.002, *p* < 0.0001; Figure 6D–H), consistent with K_ATP_ channel‐mediated hyperpolarization and subsequent dye efflux. Conversely, glibenclamide restored the fluorescence signal (0.09 ± 0.003 vs. baseline, *p* = 0.877 ± 0.14), reflecting membrane depolarization and dye accumulation. In cultured VSMCs, preconditioning with obese adiposomes attenuated the pinacidil‐induced fluorescence reduction compared to lean adiposomes (−0.714 ± 0.12 vs. –0.919 ± 0.02, *p* < 0.001), suggesting impaired K_ATP_ channel activation. Moreover, glibenclamide elicited a markedly greater fluorescence increase in the obese group (5.73 ± 1.02 vs. 0.273 ± 0.05 in lean, *p* < 0.001), further supporting the conclusion that obesity‐associated adiposomes suppress K_ATP_ channel function and promote sustained VSMC depolarization.

To validate these findings in a physiologically relevant setting, we isolated native VSMCs from lean and obese individuals and assessed membrane potential using DiBAC_4_ (3) staining. Vascular VSMCs from obese individuals displayed a significantly attenuated decrease in fluorescence in response to pinacidil (1.127 ± 0.05 vs. –0.874 ± 0.15 in lean, *p* < 0.001; Figure 6I–L), indicating impaired K_ATP_ channel‐mediated hyperpolarization. Conversely, glibenclamide elicited a markedly exaggerated increase in fluorescence in obese VSMCs (4.46 ± 0.99 vs. 0.065 ± 0.01 in lean, *p* < 0.001; Figure 6I–L), further supporting the conclusion that K_ATP_ channel activity is suppressed in obesity.

### 3.7 Obesity‐Associated Adiposomes Increase ROS Generation

**FIGURE 7:**
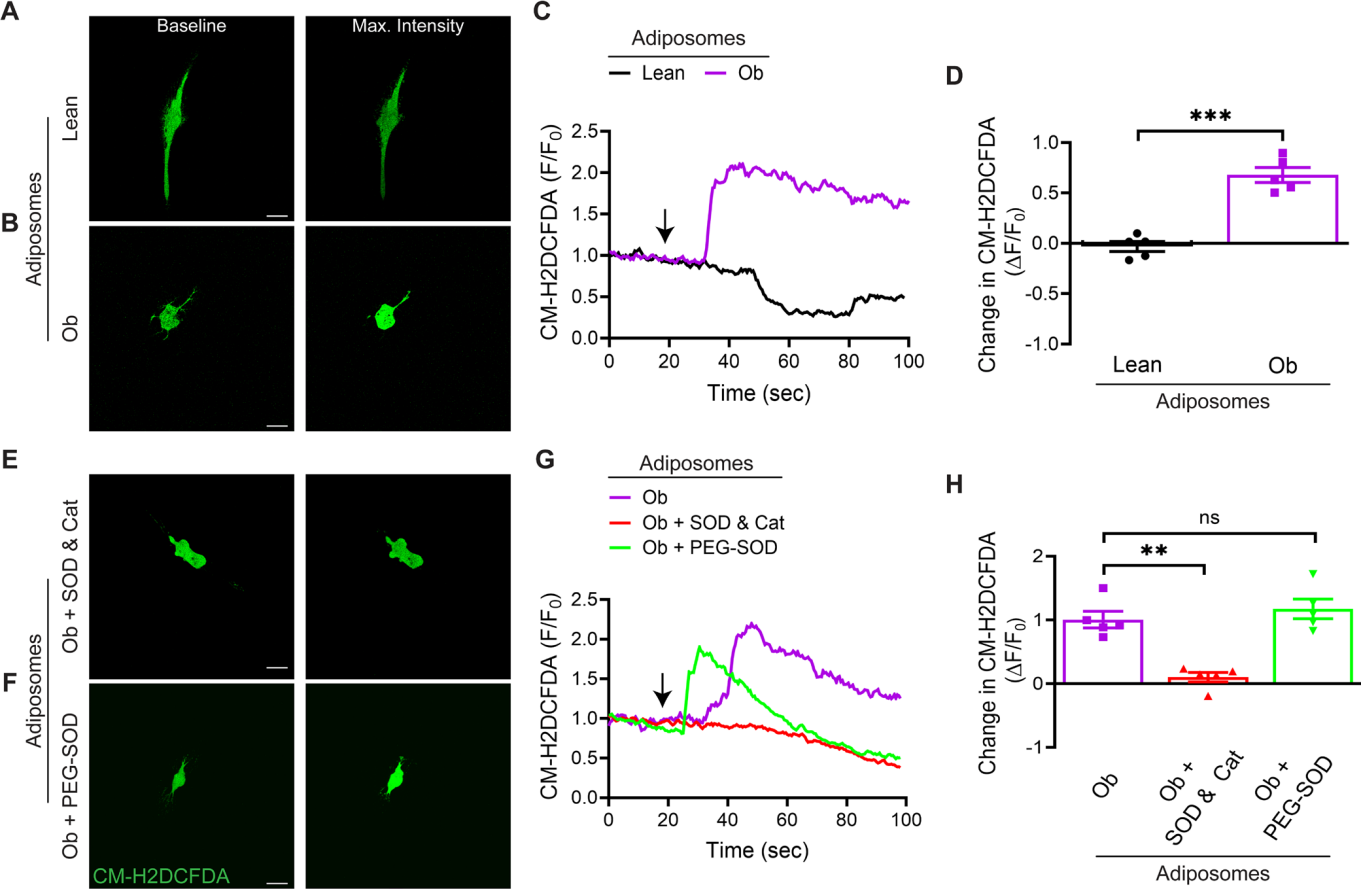
Obesity‐associated adiposomes increase ROS generation in cultured VSMCs. (A, B) Representative images of cultured VSMCs showing maximum CM‐H_2_DCFDA fluorescence, a probe for ROS, in cells treated with adiposomes from lean or obese (Ob) individuals. Fluorescence was recorded for 20 s under baseline conditions and for 80 s after exposure to adiposomes in Ca^2+^‐containing solution. Scale bar = 50 μm. (C) Representative ΔF/F₀ traces of CM‐H_2_DCFDA fluorescence over time under each condition. (D) Summary data of ΔF/F₀ changes in fluorescence intensity. Data are mean ± SEM; *n* = 5 cells per group (from independent preparations). ***p < 0.001, unpaired Student's *t* test. (E, F) Representative images of CM‐H_2_DCFDA fluorescence in VSMCs treated with obese adiposomes following pre‐incubation with superoxide dismutase (SOD; 500 U/mL) + catalase (500 U/mL) (E) or PEG‐SOD (100 U/mL) (F). Scale bar = 50 μm. (G) Representative ΔF/F₀ time‐course plots for CM‐H_2_DCFDA fluorescence under each condition. (H) Summary data of ΔF/F₀ changes in fluorescence intensity. Data are mean ± SEM; *n* = 5 cells per group (from independent preparations). **p < 0.01, two‐way ANOVA with Tukey's multiple comparisons test; ns, not significant (*p* ≥ 0.05).

We and others have previously demonstrated that adiposomes from obese individuals contain elevated levels of free fatty acids, ceramides, and other components known to promote ROS generation (Hussein et al. 2024; Blandin et al. 2023; Mirza et al. 2023). To evaluate the effect of obesity‐related adiposomes on ROS production, CM‐H_2_DCFDA, a fluorescent probe for ROS detection (Mirza et al. 2023; Metwally et al. 2024), was utilized. Exposure of cultured VSMCs to adiposomes derived from obese individuals led to a significant elevation in ROS production compared to those treated with adiposomes from lean donors (Figure 7A–D). To further identify the source of ROS production, cultured VSMCs were pre‐treated with either SOD with catalase to scavenge extracellular ROS or PEG‐SOD to target intracellular ROS. Treatment with obese adiposomes induced significant increases in CM‐H_2_DCFDA fluorescence in VSMCs, which was markedly attenuated by extracellular SOD/catalase (1.07 ± 0.21 vs. 0.095 ± 0.02, *p* = 0.003; Figure 7E–H), but unaffected by PEG‐SOD (1.075 ± 0.21 vs. 1.19 ± 0.22, *p* = 0.839; Figure 7E–H). These results suggest that adiposome‐induced ROS generation originates predominantly from extracellular superoxide radicals.

### 3.8 Extracellular ROS Scavenging Reduces Obese Adiposome‐Mediated Ca^2+^ Influx and Hypercontractility

**FIGURE 8:**
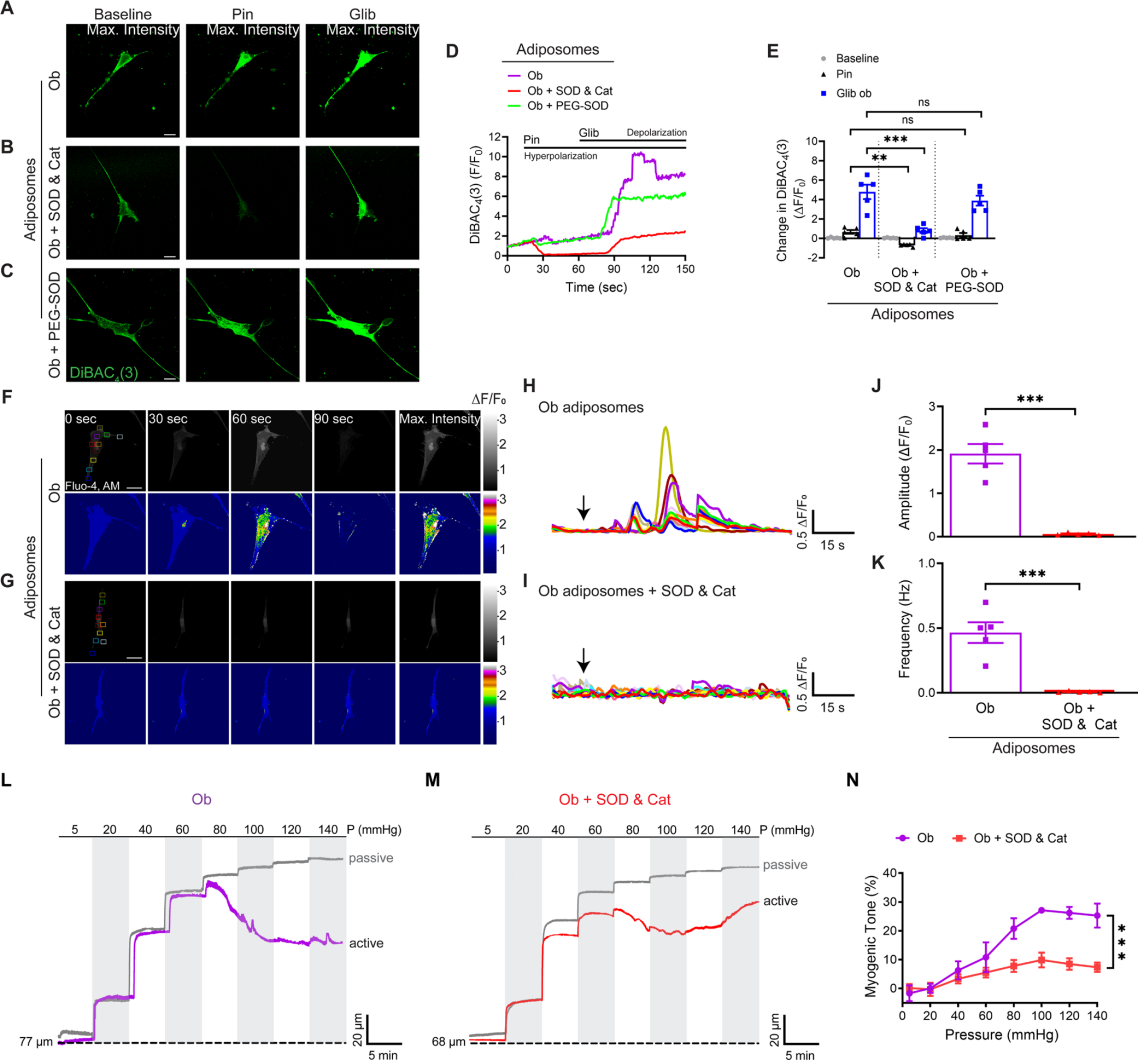
Extracellular ROS scavenger reduces obesity‐associated adiposomes‐mediated Ca^2+^ influx and hypercontractility. (A–C) Representative images of cultured VSMCs showing maximum DiBAC_4_ (3) fluorescence after treatment with adiposomes from obese individuals. Measurements were made following application of pinacidil (Pin; 10 μM) and glibenclamide (Glib; 10 μM), with or without pretreatment using superoxide dismutase (SOD; 500 U/mL) + catalase (500 U/mL) or PEG‐SOD (100 U/mL). Scale bar = 50 μm. (D) Representative F/F₀ time‐course plots of DiBAC_4_ (3) fluorescence under each condition. (E) Quantification of ΔF/F₀ changes in DiBAC_4_ (3) fluorescence intensity. Data are mean ± SEM; *n* = 5 cells per group (independent preparations). **p < 0.01, ***p < 0.001, two‐way ANOVA with Tukey's multiple comparisons test; ns, not significant (*p* ≥ 0.05). (F, G) Representative grayscale and pseudocolored images of cultured VSMCs loaded with Fluo‐4 AM and treated with obese adiposomes. Recordings were obtained under baseline conditions for 15 s, followed by 90 s exposure to adiposomes in Ca^2+^‐containing solution (2 mM Ca^2+^), with or without SOD + catalase pretreatment. Scale bar = 50 μm. (H, I) Representative ΔF/F₀ vs. time plots showing Ca^2+^ traces from multiple ROIs under the indicated conditions. (J, K) Summary data showing the amplitude (ΔF/F₀) and frequency (Hz) following adiposome treatment under the indicated conditions. Data are presented as mean ± SEM, *n* = 5 cells per group (cells were imaged from independent preparations); ***p < 0.001, unpaired Student's *t* test. (L, M) Representative tracings of intraluminal diameter changes in isolated adipose arterioles from obese individuals with or without pretreatment using SOD + catalase. (N) Summary data of myogenic tone (%). Data are mean ± SEM; *n* = 6 vessels (2 per individual, 3 individuals/group). ***p < 0.001, two‐way ANOVA with Šidák's multiple comparisons test.

To assess the contribution of ROS to the suppression of K_ATP_ channel activity by obese adiposomes, DiBAC_4_ (3) fluorescence was measured in cultured VSMCs exposed to obese adiposomes, with and without ROS scavenger treatment. Our data showed that VSMCs treated with obese adiposomes exhibited blunted DiBAC_4_ (3) fluorescence responses to pinacidil. Pretreatment with SOD and catalase (0.677 ± 0.11 vs. −0.719 ± −0.12, *p* = 0.001; Figure 8A–E), but not PEG‐SOD (0.6773 ± 0.05 vs. 0.424 ± 0.1, *p* = 0.514; Figure 8A–E), restored the pinacidil‐induced fluorescence decrease. Similarly, pretreatment with SOD and catalase normalized the glibenclamide‐induced fluorescence increase (5.395 ± 1.01 vs. 0.840 ± 0.12, *p* > 0.001; Figure 8A–E), while PEG‐SOD had no effect (5.395 ± 1.02 vs. 3.960 ± 0.92, *p* = 0.141; Figure 8A–E). These findings demonstrate that obesity‐associated adiposomes suppress K_ATP_ channel function primarily through extracellular ROS‐dependent mechanisms.

To assess the role of extracellular ROS in mediating Ca^2+^ influx, cultured VSMCs were treated with obese adiposomes together with SOD and catalase. Obese adiposome treatment significantly increased intracellular Ca^2+^ amplitude (1.91 ± 0.23 vs. 0.063 ± 0.02 adiposome alone, *p* < 0.001) and frequency (0.465 ± 0.03 vs. 0.0082 ± 0.001 adiposome alone, *p* < 0.001), an effect that was markedly attenuated by SOD/catalase pretreatment (Figure 8F–K). Complementary translational experiments demonstrated that treatment with SOD/catalase effectively attenuated the hypercontractile response of adipose arterioles from obese donors (*p* < 0.001; Figure 8L–N). Together, these findings implicate extracellular ROS as key mediators of adiposome‐induced Ca^2+^ dysregulation in VSMCs, contributing to the vascular hypercontractility observed in obesity.

### 3.9 Ceramide Depletion Attenuates Adiposome‐Induced Hypercontractility

Our previous studies and others have demonstrated that adiposomes isolated from obese individuals are enriched with ceramide, a bioactive lipid molecule that plays critical roles in cellular signaling pathways but may induce detrimental effects on recipient cells when present in excess levels (Hussein et al. 2024; Blandin et al. 2023; Mirza et al. 2023). Ceramide's vasoactive properties have been studied in various vascular preparations, where it induces sustained vasoconstriction (Cogolludo et al. 2019; Zheng et al. 2000), and promotes ROS production, further enhancing vasoconstrictive responses (Moral‐Sanz et al. 2011; Frazziano et al. 2011). This study examined whether depleting ceramides from obese adiposomes could mitigate their pro‐contractile effects on vascular function.

**FIGURE 9:**
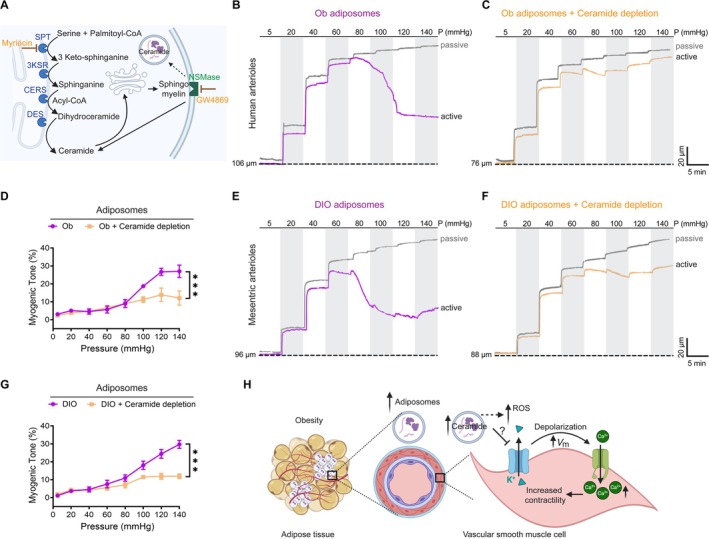
Ceramide depletion attenuates adiposome‐induced hypercontractility. (A) Schematic of the experimental strategy for ceramide depletion. Ceramides are synthesized via the de novo pathway through sequential enzymatic reactions or generated from sphingomyelin by neutral sphingomyelinase (N‐SMase). Ceramide levels were reduced using Myriocin, a serine palmitoyltransferase (SPT) inhibitor that blocks de novo ceramide synthesis, together with GW4869, a noncompetitive N‐SMase inhibitor. (B, C) representative tracings of intraluminal diameter changes in isolated human arterioles from healthy controls after incubation with adiposomes or ceramide‐depleted adiposomes derived from obese individuals. (D) Quantification of myogenic tone (%). Data are mean ± SEM; *n* = 6 vessels from 3 individuals. ***p < 0.001, two‐way ANOVA with Šidák's multiple comparisons test. (E, F) representative tracings of mesenteric arteriole diameter changes in SD mice after incubation with adiposomes or ceramide‐depleted adiposomes from DIO mice. (G) Quantification of myogenic tone (%) in mesenteric arterioles. Data are mean ± SEM; *n* = 6 vessels from 3 mice. ***p < 0.001, two‐way ANOVA with Šidák's test. (H) Proposed mechanistic model showing that obesity‐associated adiposomes, enriched in ceramides, promote ROS generation. Elevated ROS inhibits K_ATP_ channel activity, leading to membrane depolarization, enhanced Ca^2+^ influx, and hypercontractility of VSMCs.

Ceramide biosynthesis occurs through either the de novo pathway SPT or sphingomyelin hydrolysis via N‐SMase (Cogolludo et al. 2019). To reduce ceramide levels, we used myriocin (an SPT inhibitor that blocks de novo synthesis) combined with GW4869 (a noncompetitive N‐SMase inhibitor) (Figure 9A). Interestingly, ceramide‐depleted adiposomes isolated from obese individuals significantly reduced vasoconstriction compared to non‐depleted adiposomes (*p* < 0.001; Figure 9B–D). Similarly, isolated mesenteric (Figure 9E–G) and skeletal muscle arterioles (Figure S6) from SD mice were co‐incubated with native or ceramide‐depleted adiposomes from DIO mice. Ceramide‐depleted DIO adiposomes significantly attenuated vasoconstriction compared to native DIO adiposomes (*p* < 0.001; Figure 9E–G; Figure S6), demonstrating that ceramide is a key mediator of obese adiposome‐induced vascular hypercontractility.

Collectively, our findings support a mechanistic model (Figure 9H) in which ceramide‐enriched adiposomes from obese individuals drive vascular dysfunction via a three‐step cascade: (1) induction of excessive ROS production, (2) inhibition of K_ATP_ channel activity in VSMCs (though the precise mechanism by which ceramide inhibits K_ATP_ channels remains unknown and requires further investigation), and (3) membrane depolarization that triggers pathological Ca^2+^ influx and sustained hypercontractility. This sequence elevates basal vascular tone and may clinically contribute to increased blood pressure, impaired tissue perfusion, and the progression of obesity‐associated cardiovascular complications.

## 4 Discussion

This study uncovers a novel and clinically significant mechanism linking obesity‐associated adiposomes to microvascular dysfunction. We demonstrate that adiposomes derived from obese humans and DIO mice enhance VSMCs contractility, directly contributing to exaggerated vasoconstriction in small resistance arterioles. At the cellular level, these adiposomes amplify pathological Ca^2+^ influx in VSMCs by suppressing K_ATP_ channel activity, driven primarily by elevated ROS generation. These findings position extracellular‐vesicle signaling as a critical conduit of adipose–vascular crosstalk, transforming local adipose dysfunction into a systemic vascular threat. Crucially, pharmacological scavenging of ROS normalizes Ca^2+^ signaling and reverses vascular hypercontractility, while ceramide depletion substantially mitigates the pro‐contractile effects of adiposomes. By demonstrating that adiposome cargo alters VSMCs excitability, we reveal how metabolic stress in adipose tissue can remotely reprogram vascular tone, an archetype of inter‐organ communication gone awry. Collectively, our findings identify a ceramide/ROS/K_ATP_ channel signaling axis as a central pathway underlying obesity‐induced microvascular dysfunction, offering new therapeutic targets for managing obesity‐related hypertension and associated vascular complications.

Previous findings from our group and others have shown that obesity significantly promotes endothelial dysfunction, a major contributor to hypertension and cardiovascular morbidity (Mahmoud et al. 2016; Ali et al. 2021; Stapleton et al. 2008). Obesity is consistently associated with impaired vasoreactivity, elevated oxidative stress, and chronic inflammation, core mechanisms of obesity‐related CVDs (Powell‐Wiley et al. 2021). In line with this, our current study demonstrates markedly increased pressure‐induced vasoconstriction in arterioles from obese individuals, emphasizing the clinical relevance of targeting microvascular hypercontractility. Using pressure myography, we observed significantly elevated myogenic tone, accompanied by amplified Ca^2+^ influx in VSMCs that persisted after ER Ca^2+^ depletion, confirming reliance on extracellular Ca^2+^ entry. Despite this hypercontractility, obese arterioles exhibited increased passive diameter and distensibility, likely reflecting outward remodeling to reduce stiffness. However, this structural adaptation failed to normalize tone, indicating a decoupling of structural and functional responses.

Dysfunctional adipose tissue exhibits enhanced infiltration of macrophages and dendritic cells, elevated secretion of pro‐inflammatory adipokines and cytokines (e.g., resistin, visfatin, leptin, MCP‐1, TNF‐α, IL‐6), and diminished production of the anti‐inflammatory adipokine adiponectin (Valenzuela et al. 2023; Sakers et al. 2022). These factors act on VSMCs to alter vascular tone and promote contractile, proliferative, and migratory responses. Key signaling pathways driving adipose‐induced phenotypic switching in VSMCs include ERK, p38 MAPK, cAMP‐ERK, and TGF‐β1/ERK cascades. Additionally, VSMC dysfunction is exacerbated by OPN signaling, PKCε‐dependent NADPH oxidase (Nox) activation, ROS generation, and AP‐1‐mediated transcription (Chang et al. 2020). Despite these insights, most studies have focused on adipose‐derived soluble factors, leaving a critical gap in understanding the role of adiposomes.

Excess visceral fat is a major driver of cardiovascular and metabolic complications, not just due to fat mass but also its dysfunctional nature. In obesity, visceral fat becomes highly inflamed, oxidatively stressed, and metabolically dysregulated (Farb and Gokce 2015). These disturbances cause substantial damage to local adipose tissue vasculature and exert profound systemic effects, impairing remote and peripheral blood vessels and dramatically increasing the cardiovascular risks associated with obesity. Our findings show that dysfunctional visceral fat in obese individuals produces significantly more adiposomes than in lean controls, with lipid cargo enriched in short‐chain, saturated ceramides (Hussein et al. 2024; Mahmoud et al. 2025; Mirza et al. 2023; Mirza, Hassan, et al. 2022; Mirza, Naquiallah, et al. 2022). Building on this, our study uncovers how these adiposomes disrupt vascular health by altering VSMC calcium signaling and promoting hypercontractility, key drivers of adverse vascular outcomes. Our data therefore extend the concept of organ crosstalk beyond the classic endothelium–myocyte axis to include a dynamic “adipose–extracellular vesicles–vessel” loop that amplifies cardiometabolic risk in obesity.

Few studies have examined adiposome effects on VSMCs. Li et al. (2019) showed that visceral adiposomes from obese mice induced a contractile‐to‐synthetic phenotypic shift in aortic VSMCs, driven by elevated miR‐221‐p3. However, the impact of adiposomes on microvascular SMCs, key regulators of peripheral resistance and hypertension, remains largely unexplored. Our previous work showed that obese adiposomes disrupt caveolae, impair eNOS activity, and compromise endothelial integrity (Mirza et al. 2023; SenthilKumar et al. 2024). Our findings reveal that adiposomes derived from obese individuals not only impair endothelial function but also directly augment vascular smooth muscle cell (VSMC) contractility. This dual mechanism suggests that vascular hypercontractility may arise from endothelial dysfunction, intrinsic VSMC effects, or their synergistic interaction, thereby establishing adipose tissue abnormalities as a critical driver of systemic vascular pathology.

Additionally, our results indicate that obesity‐altered adiposomes do not directly impair intrinsic VGCC function, as evidenced by the preserved responses to depolarization induced by high extracellular K^+^ in our experimental models. This suggests the observed hypercontractility arises upstream of VGCC activation, primarily through changes in membrane potential and ROS‐mediated signaling pathways. In particular, intracellular Ca^2+^ regulation in VSMCs is tightly governed by membrane potential via K_ATP_ channels (Brayden 2002), and dysfunction of these channels has been implicated in hypertension, metabolic syndrome, and diabetes‐related vascular complications (Nichols et al. 2013; Olson and Terzic 2010). Consistent with this, our findings show that adiposomes from obese individuals attenuate K_ATP_‐mediated hyperpolarization and enhance depolarization‐induced Ca^2+^ influx, thereby providing a mechanistic explanation for how adiposomes promote vascular hypercontractility. These observations align with previous studies, including Climent et al. (2014), which showed that enhanced Ca^2+^ influx in obesity can occur independently of VGCC activity due to alterations in membrane potential, as well as in vivo studies where diet‐induced obesity reduced K_ATP_ current density and relaxation responses in VSMCs (Fan et al. 2009). Importantly, our study advances this literature by directly linking adiposomal signaling to K_ATP_ channel dysfunction.

We acknowledge that the precise ion channels mediating this extracellular Ca^2+^ entry were not delineated in the current study. In addition to voltage‐gated Ca^2+^ channels (VGCCs), non‐VGCC sources, particularly transient receptor potential (TRP) channels, may contribute to the observed VGCC‐independent hypercontractility. For example, TRPC6 channels are activated downstream of mechanosensitive and Gq‐coupled GPCR signaling through diacylglycerol (DAG), while TRPV1 channels can also be engaged through GqPCR‐dependent pathways. Both TRPC6 and TRPV1 are capable of facilitating Ca^2+^ entry and promoting vasoconstriction (Davis et al. 2023; Solano et al. 2024), and thus represent plausible candidates for mediating adiposome effects in VSMCs.

The role of ROS in obesity‐related vascular pathology is well‐established, as oxidative stress alters vascular cell structure and function, contributing to inflammation, endothelial dysfunction, and impaired vasodilation (Zhou et al. 2021). Our imaging data prove ROS are key mediators of adiposome‐induced K_ATP_ channel dysfunction. Specifically, scavenging extracellular ROS attenuated Ca^2+^ dysregulation and hypercontractility triggered by obese adiposomes. These findings align with recent evidence that free radicals influence vascular cell growth, migration, inflammation, decreased NO bioavailability, and extracellular matrix (ECM) deposition. Elevated O_2_^−^ levels observed in the aortas of obese rats are closely tied to vascular inflammation and aortic fibrosis (Martinez‐Martinez et al. 2021). Our study uniquely positions ROS at the intersection of adiposome signaling and VSMC dysfunction, reinforcing its relevance as a therapeutic target in obesity‐induced vascular complications.

Ceramide accumulation, a hallmark of obesity‐related adipose dysfunction, is recognized as a key mediator of cardiovascular disease due to its pro‐inflammatory and vasoactive effects (Freed et al. 2014; Chaurasia et al. 2020). Seminal work by McGurk et al. (2021) established a robust association between high circulating ceramide levels and increased cardiovascular risk, while Akhiyat et al. (2022) further demonstrated ceramide‐mediated impairment of endothelial function. Consistently, our recent work has shown that obesity‐associated adiposomes are enriched in ceramides and other bioactive lipids, along with reduced levels of phospholipids and sphingomyelins, compared to those from lean donors (Mahmoud et al. 2025). Notably, ceramides emerged as the top differential lipid species in EVs between obese and lean individuals, underscoring their central role in obesity‐related adiposome remodeling. These compositional changes suggest that ceramides and related lipids may directly impair vascular cell function. In the present study, we focused on ceramides given their established role as a hallmark of adipose dysfunction and their feasibility as a mechanistic target. Our findings show that ceramide‐enriched obese adiposomes directly drive vascular hypercontractility, and importantly, that ceramide depletion blunted these effects, underscoring the therapeutic potential of targeting ceramide synthesis. These results resonate with growing evidence supporting ceramide reduction as a compelling strategy to lower cardiovascular risk in the context of metabolic syndrome and obesity (Wang et al. 2025; Aburasayn et al. 2016). Because sphingosine‐1‐phosphate (S1P) often counteracts ceramide's vascular effects (Piccoli et al. 2023), the balance between S1P and ceramide (S1P/Cer ratio) may better predict EV‐induced vascular dysfunction than ceramide alone. Therefore, future studies should quantify both lipids and broaden profiling to identify other bioactive mediators. Recent clinical studies underscore the relevance of circulating ceramide remodeling in cardiometabolic disease. Specific ceramide species have been identified as predictors of cardiovascular risk years before disease onset. Importantly, therapies such as GLP‐1 receptor agonists (GLP‐1RAs) and SGLT2 inhibitors not only improve glycemic control but also lower circulating ceramides in patients with type 2 diabetes (Shalaby et al. 2025; Wretlind et al. 2023; Denimal et al. 2023). In the context of our results, this raises the intriguing possibility that therapeutic modulation of EV sphingolipid cargo, including the ceramide/S1P balance, could represent a novel mechanism linking pharmacologic interventions to improved vascular function and blood pressure regulation.

Our study has important implications for understanding obesity‐associated hypertension, a highly prevalent cardiovascular disorder. We identify ceramide‐ and ROS‐dependent suppression of K_ATP_ channels by obesity‐related adiposomes as a key mechanism driving elevated vascular tone and systemic resistance, establishing a direct pathophysiological link between obesity and hypertension. These mechanistic insights align with clinical observations by Davy and Hall (2004), who emphasized the role of microvascular dysfunction and increased vascular tone in hypertensive individuals with obesity. Similarly, studies in Ossabaw swine demonstrated that obese coronary PVAT selectively inhibits K_ATP_‐mediated dilation without altering channel expression (Noblet et al. 2015), indicating that adipose‐derived factors can directly compromise channel function, though the precise mechanism by which ceramide inhibits K_ATP_ channels remains unknown and requires further investigation. Targeting this maladaptive adipose‐to‐vessel dialogue may break the vicious cycle linking metabolic inflammation to heightened peripheral resistance and hypertension.

In conclusion, our findings advance our understanding of how obesity‐associated adiposomes worsen vascular dysfunction by impairing K_ATP_ channels and disrupting Ca^2+^ homeostasis in VSMCs. ROS and ceramides emerge as key mediators, presenting promising therapeutic targets for obesity‐related cardiovascular complications. Future studies should focus on translating these insights into effective treatments for cardiovascular disease in obesity and metabolic syndrome.

While our study provides compelling evidence linking obesity‐associated adiposomes to microvascular dysfunction, several limitations warrant consideration. Although we used human and DIO mouse samples, the cross‐sectional design and small cohort sizes may limit generalizability. We also focused primarily on visceral adipose tissue, which may not capture the full extent of adiposome heterogeneity across fat depots. Mechanistically, our work centers on ROS generation, ceramide enrichment, and K_ATP_ channel modulation; however, other factors, such as other lipid metabolites, ion channels, or paracrine signals, may also contribute to VSMC dysfunction. Moreover, while ex vivo pressure myography and cell‐based assays provide detailed functional insights, they do not fully replicate complex in vivo interactions. A key limitation of our work is that it primarily addresses the acute effects of adiposomes on vascular tone. While obese arterioles exhibited hypercontractility with evidence of outward remodeling, acute adiposome exposure increased contractility without altering passive diameter or distensibility, suggesting purely functional changes. It remains unclear whether chronic adiposome signaling drives structural remodeling and sustained vascular dysfunction. Future in vivo studies are therefore required to determine whether these vesicles merely maintain acute effects or promote long‐term adaptations that exacerbate cardiovascular risk in obesity. Lastly, although our findings point to promising therapeutic targets, further in vivo studies and clinical trials are necessary to evaluate the efficacy, safety, and long‐term benefits of modulating these pathways in obese populations.

## Author Contributions

A.M.M. supervised the project. E.M. and A.M.M. designed the experiments. I.M., M.H.M., and A.M.M. manage recruiting patients for this study. F.M.B., C.H., and M.H.M. assist in surgical procedures. E.M. performed and analyzed pressure myography experiments. E.M. and I.M. performed Ca^2+^, ROS, and DiBAC_4_ (3) imaging experiments. E.M. and A.M.M. analyzed the data. The initial manuscript draft and figures were prepared by E.M., S.M.M., and A.M.M. E.M., I.M., M.H.M., P.C.R., I.L., U.J.R., B.T.L., and A.M.M. revised and approved the manuscript. No artificial intelligence tools were used in the preparation of this work.

## Conflicts of Interest

The authors declare no conflicts of interest.

## Supporting information


**Figure S1:** Small skeletal muscle arterioles from DIO mice are hypercontractile. (A) Isolation of a Small skeletal muscle arterioles (arrow) from mice. (B, C) Representative tracings showing changes in the intraluminal diameter of isolated skeletal muscle arterioles from SD and DIO mice. (D) Summary data showing the percentage of myogenic tone in skeletal muscle arterioles from SD and DIO mice. Data are presented as mean ± SEM, *n* = 6 vessels from 3 mice; *p < 0.05, two‐way ANOVA with Šidák's multiple comparisons test. (E, F) Summary data illustrating the passive inner diameter (μm) and distensibility coefficient of isolated skeletal muscle arterioles. Data are presented as mean ± SEM, *n* = 6 vessels from 3 mice per group, two‐way ANOVA followed by Šidák's multiple comparisons test, ns: not significant, *p* ≥ 0.05. (G) Summary data show the constriction of isolated skeletal muscle arterioles from the indicated mice in response to 60 mM KCl. Data are presented as mean ± SEM, *n* = 6 arteries, unpaired Student's *t* test, ns: not significant; *p* ≥ 0.05.
**Figure S2:** Adiposomes exert their effects on vSMCs independently of endothelial cells. (A, B) Summary data showing the percentage of myogenic tone in mesenteric arterioles isolated from SD mice. Arterioles were incubated with adiposomes derived from DIO mice, either alone or in combination with the nitric oxide donor sodium nitroprusside (SNP) (10 μM) (A) or following endothelial denudation by gentle air perfusion (B). Data are presented as mean ± SEM; *p < 0.05, two‐way ANOVA with Šidák's multiple comparisons test (*n* = 3 vessels from 3 mice).
**Figure S3:** Adiposome uptake in intact vessels. (A–C) Representative images of whole‐mount mesenteric blood vessels treated with adiposomes isolated from either SD or DIO mice. Adiposome uptake is visualized using BODIPY‐labeled particles (red, arrows). vSMCs are identified by α‐smooth muscle actin (α‐SMA, green), and nuclei are counterstained with DAPI (blue). Scale bar = 50 μm.
**Figure S4:** Adiposome uptake with cultured vSMCs. (A) Representative image of adiposome uptake labeled with BODIPY dye (red, arrows) by vSMCs (cell membrane labeled green [phalloidin] and nuclei labeled blue [DAPI]). Scale bar = 50 μm. (B) Summary data showing red fluorescent signal intensity expressed in arbitrary units (AU) in response to adiposome treatment isolated from lean and obese individuals. Data are presented as mean ± SEM, *n* = 5 cells per group (cells were imaged from independent preparations), ***p < 0.001, unpaired Student's *t* test.
**Figure S5:** DIO mice adiposomes increase Ca^2+^ influx in cultured vSMCs. (A–D) Representative grayscale and pseudocolored images of cultured vSMCs treated with adiposomes isolated from SD and DIO mice under different conditions. Cells were stained with Fluo‐4 AM. Recordings were initially made under baseline conditions for 30 s, followed by exposure to adiposomes in a Ca^2+^‐containing solution or pretreatment with thapsigargin (TG) and a Ca^2+^‐free solution for 120 s. Colored boxes highlight ROIs with active Ca^2+^ signals. Scale bar = 50 μm. (E–H) Representative ΔF/F₀ vs. time plots showing Ca^2+^ traces from multiple ROIs under the indicated conditions. (I–K) Summary data showing the amplitude (ΔF/F₀), frequency (Hz), and signal duration in SMCs. Data are presented as mean ± SEM, *n* = 5 cells per group (cells were imaged from independent preparations); **p < 0.01; ***p < 0.001, two‐way ANOVA with Tukey's multiple comparisons test; ns: not significant, *p* ≥ 0.05.
**Figure S6:** Ceramide depletion attenuates adiposome‐mediated hypercontractility in skeletal muscle arterioles. (A, B) Representative tracings showing changes in the intraluminal diameter of isolated skeletal muscle arterioles from SD mice following co‐incubation with adiposomes or ceramide‐depleted adiposomes isolated from DIO mice. (C) Summary data quantifying the percentage of myogenic tone. Data are expressed as mean ± SEM, *n* = 3 vessels from 3 mice; ***p < 0.001, two‐way ANOVA with Šidák's multiple comparisons test.
**Table S1:** Clinical characteristics of lean vs. obese participants.

**Video S1:** Ca^2+^ signal in isolated native vSMCs from a lean individual, recorded in the presence of ACh (5 μM).

**Video S2:** Spontaneous and ACh‐evoked Ca^2+^ events in isolated native vSMCs isolated from an obese individual. Recordings show intracellular Ca^2+^ dynamics following stimulation with ACh (5 μM).

**Video S3:** Pretreatment with thapsigargin (TG, 2 μM) did not abolish spontaneous ACh‐evoked Ca^2+^ events in isolated native vSMCs isolated from an obese individual.

**Video S4:** Ca^2+^ signaling in cultured vSMCs following exposure to adiposomes derived from a lean individual in the presence of 2 mM extracellular Ca^2+^.

**Video S5:** Ca^2+^ signaling in cultured vSMCs following exposure to adiposomes derived from an obese individual in the presence of 2 mM extracellular Ca^2+^.

**Video S6:** Ca^2+^ signaling in cultured vSMCs following exposure to adiposomes derived from an obese individual in the presence of 0 mM extracellular Ca^2+^.

**Video S7:** Ca^2+^ signaling in cultured vSMCs following exposure to adiposomes derived from SD mice in the presence of 2 mM extracellular Ca^2+^.

**Video S8:** Ca^2+^ signaling in cultured vSMCs following exposure to adiposomes derived from DIO mice in the presence of 2 mM extracellular Ca^2+^.

**Video S9:** Ca^2+^ signaling in cultured vSMCs following exposure to adiposomes derived from DIO mice in the presence of 2 mM extracellular Ca^2+^ and Pretreatment with thapsigargin (TG, 2 μM).

**Video S10:** Ca^2+^ signaling in cultured vSMCs following exposure to adiposomes derived from DIO mice in the presence of 0 mM extracellular Ca^2+^.

## Data Availability

The data that support the findings of this study are available from the corresponding author upon reasonable request.

## References

[cph470053-bib-0001] Aburasayn, H. , R. Al Batran , and J. R. Ussher . 2016. “Targeting Ceramide Metabolism in Obesity.” American Journal of Physiology. Endocrinology and Metabolism 311: E423–E435. 27382035 10.1152/ajpendo.00133.2016

[cph470053-bib-0002] Akhiyat, N. , V. Vasile , A. Ahmad , et al. 2022. “Plasma Ceramide Levels Are Elevated in Patients With Early Coronary Atherosclerosis and Endothelial Dysfunction.” Journal of the American Heart Association 11: e022852. 35301857 10.1161/JAHA.121.022852 PMC9075496

[cph470053-bib-0003] Ali, M. M. , C. Hassan , M. Masrur , et al. 2021. “Adipose Tissue Hypoxia Correlates With Adipokine Hypomethylation and Vascular Dysfunction.” Biomedicine 9, no. 8: 1034. 10.3390/biomedicines9081034 PMC8394952 34440238

[cph470053-bib-0004] Aziz, Q. , A. M. Thomas , J. Gomes , et al. 2014. “The Atp‐Sensitive Potassium Channel Subunit, kir6.1, in Vascular Smooth Muscle Plays a Major Role in Blood Pressure Control.” Hypertension 64: 523–529. 24914196 10.1161/HYPERTENSIONAHA.114.03116

[cph470053-bib-0005] Blandin, A. , I. Dugail , G. Hilairet , et al. 2023. “Lipidomic Analysis of Adipose‐Derived Extracellular Vesicles Reveals Specific Ev Lipid Sorting Informative of the Obesity Metabolic State.” Cell Reports 42 no. 3: 112169. 36862553 10.1016/j.celrep.2023.112169

[cph470053-bib-0006] Brayden, J. E. 2002. “Functional Roles of K_ATP_ Channels in Vascular Smooth Muscle.” Clinical and Experimental Pharmacology and Physiology 29: 312–316. 11985542 10.1046/j.1440-1681.2002.03650.x

[cph470053-bib-0007] Chait, A. , and L. J. den Hartigh . 2020. “Adipose Tissue Distribution, Inflammation and Its Metabolic Consequences, Including Diabetes and Cardiovascular Disease.” Frontiers in Cardiovascular Medicine 7: 22. 32158768 10.3389/fcvm.2020.00022 PMC7052117

[cph470053-bib-0008] Chang, L. , M. T. Garcia‐Barrio , and Y. E. Chen . 2020. “Perivascular Adipose Tissue Regulates Vascular Function by Targeting Vascular Smooth Muscle Cells.” Arteriosclerosis, Thrombosis, and Vascular Biology 40: 1094–1109. 32188271 10.1161/ATVBAHA.120.312464 PMC7441816

[cph470053-bib-0009] Chaurasia, B. , C. L. Talbot , and S. A. Summers . 2020. “Adipocyte Ceramides‐The Nexus of Inflammation and Metabolic Disease.” Frontiers in Immunology 11: 576347. 33072120 10.3389/fimmu.2020.576347 PMC7538607

[cph470053-bib-0010] Climent, B. , L. Moreno , P. Martinez , et al. 2014. “Upregulation of sk3 and ik1 Channels Contributes to the Enhanced Endothelial Calcium Signaling and the Preserved Coronary Relaxation in Obese Zucker Rats.” PLoS One 9: e109432. 25302606 10.1371/journal.pone.0109432 PMC4193814

[cph470053-bib-0011] Cogolludo, A. , E. Villamor , F. Perez‐Vizcaino , and L. Moreno . 2019. “Ceramide and Regulation of Vascular Tone.” International Journal of Molecular Sciences 20: 411. 30669371 10.3390/ijms20020411 PMC6359388

[cph470053-bib-0012] Dabertrand, F. , M. T. Nelson , and J. E. Brayden . 2012. “Acidosis Dilates Brain Parenchymal Arterioles by Conversion of Calcium Waves to Sparks to Activate Bk Channels.” Circulation Research 110: 285–294. 22095728 10.1161/CIRCRESAHA.111.258145 PMC3505882

[cph470053-bib-0013] Dart, C. 2014. “Verdict in the Smooth Muscle K_ATP_ Channel Case.” Hypertension 64: 457–458. 24914201 10.1161/HYPERTENSIONAHA.114.03289

[cph470053-bib-0014] Davis, M. J. , S. Earley , Y. S. Li , and S. Chien . 2023. “Vascular Mechanotransduction.” Physiological Reviews 103: 1247–1421. 36603156 10.1152/physrev.00053.2021 PMC9942936

[cph470053-bib-0015] Davy, K. P. , and J. E. Hall . 2004. “Obesity and Hypertension: Two Epidemics or One?” American Journal of Physiology. Regulatory, Integrative and Comparative Physiology 286: R803–R813. 15068965 10.1152/ajpregu.00707.2003

[cph470053-bib-0016] Denimal, D. , V. Bergas , J. P. Pais‐de‐Barros , et al. 2023. “Liraglutide Reduces Plasma Dihydroceramide Levels in Patients With Type 2 Diabetes.” Cardiovascular Diabetology 22: 104. 37143040 10.1186/s12933-023-01845-0 PMC10158384

[cph470053-bib-0017] Fan, L. H. , H. Y. Tian , M. L. Yang , et al. 2009. “High‐Fat Diet May Impair k(Atp) Channels in Vascular Smooth Muscle Cells.” Biomedicine & Pharmacotherapy 63: 165–170. 18339514 10.1016/j.biopha.2008.01.005

[cph470053-bib-0018] Farb, M. G. , and N. Gokce . 2015. “Visceral Adiposopathy: A Vascular Perspective.” Hormone Molecular Biology and Clinical Investigation 21, no. 2: 125–136. 25781557 10.1515/hmbci-2014-0047 PMC4442778

[cph470053-bib-0019] Frazziano, G. , L. Moreno , J. Moral‐Sanz , et al. 2011. “Neutral Sphingomyelinase, NADPH Oxidase and Reactive Oxygen Species. Role in Acute Hypoxic Pulmonary Vasoconstriction.” Journal of Cellular Physiology 226: 2633–2640. 21792922 10.1002/jcp.22611

[cph470053-bib-0020] Freed, J. K. , A. M. Beyer , J. A. LoGiudice , J. C. Hockenberry , and D. D. Gutterman . 2014. “Ceramide Changes the Mediator of Flow‐Induced Vasodilation From Nitric Oxide to Hydrogen Peroxide in the Human Microcirculation.” Circulation Research 115: 525–532. 24920698 10.1161/CIRCRESAHA.115.303881 PMC4640193

[cph470053-bib-0021] Hagberg, C. E. , and K. L. Spalding . 2024. “White Adipocyte Dysfunction and Obesity‐Associated Pathologies in Humans.” Nature Reviews. Molecular Cell Biology 25: 270–289. 38086922 10.1038/s41580-023-00680-1

[cph470053-bib-0022] Han, Y. , S. Ye , and B. Liu . 2024. “Roles of Extracellular Vesicles Derived From Healthy and Obese Adipose Tissue in Inter‐Organ Crosstalk and Potential Clinical Implication.” Frontiers in Endocrinology 15: 1409000. 39268243 10.3389/fendo.2024.1409000 PMC11390393

[cph470053-bib-0023] Holland, W. L. , R. A. Miller , Z. V. Wang , et al. 2011. “Receptor‐Mediated Activation of Ceramidase Activity Initiates the Pleiotropic Actions of Adiponectin.” Nature Medicine 17: 55–63. 10.1038/nm.2277 PMC3134999 21186369

[cph470053-bib-0024] Hussein, M. , I. Mirza , M. Morsy , et al. 2024. “Comparison of Adiposomal Lipids Between Obese and Non‐Obese Individuals.” Metabolites 14: 464. 39195560 10.3390/metabo14080464 PMC11356626

[cph470053-bib-0025] Jackson, W. F. 2021. “Myogenic Tone in Peripheral Resistance Arteries and Arterioles: The Pressure Is on!” Frontiers in Physiology 12: 699517. 34366889 10.3389/fphys.2021.699517 PMC8339585

[cph470053-bib-0026] Koenen, M. , M. A. Hill , P. Cohen , and J. R. Sowers . 2021. “Obesity, Adipose Tissue and Vascular Dysfunction.” Circulation Research 128: 951–968. 33793327 10.1161/CIRCRESAHA.121.318093 PMC8026272

[cph470053-bib-0027] Landsberg, L. , L. J. Aronne , L. J. Beilin , et al. 2013. “Obesity‐Related Hypertension: Pathogenesis, Cardiovascular Risk, and Treatment—A Position Paper of the the Obesity Society and the American Society of Hypertension.” Obesity 21: 8–24. 23401272 10.1002/oby.20181

[cph470053-bib-0028] Le Lay, S. , S. Rome , X. Loyer , and L. Nieto . 2021. “Adipocyte‐Derived Extracellular Vesicles in Health and Diseases: Nano‐Packages With Vast Biological Properties.” FASEB Bioadvances 3: 407–419. 34124596 10.1096/fba.2020-00147 PMC8171308

[cph470053-bib-0029] Li, X. , L. L. Ballantyne , Y. Yu , and C. D. Funk . 2019. “Perivascular Adipose Tissue–Derived Extracellular Vesicle mir‐221‐3p Mediates Vascular Remodeling.” FASEB Journal 33: 12704–12722. 31469602 10.1096/fj.201901548R PMC6902668

[cph470053-bib-0030] Mahmoud, A. M. , I. Mirza , E. Metwally , et al. 2025. “Lipidomic Profiling of Human Adiposomes Identifies Specific Lipid Shifts Linked to Obesity and Cardiometabolic Risk.” JCI Insight 10: e191872. 40548377 10.1172/jci.insight.191872 PMC12226050

[cph470053-bib-0031] Mahmoud, A. M. , M. R. Szczurek , B. K. Blackburn , et al. 2016. “Hyperinsulinemia Augments Endothelin‐1 Protein Expression and Impairs Vasodilation of Human Skeletal Muscle Arterioles.” Physiological Reports 4: e12895. 27796268 10.14814/phy2.12895 PMC5002909

[cph470053-bib-0032] Martinez‐Martinez, E. , F. V. Souza‐Neto , S. Jimenez‐Gonzalez , and V. Cachofeiro . 2021. “Oxidative Stress and Vascular Damage in the Context of Obesity: The Hidden Guest.” Antioxidants (Basel) 10: 406. 33800427 10.3390/antiox10030406 PMC7999611

[cph470053-bib-0033] McGurk, K. A. , B. D. Keavney , and A. Nicolaou . 2021. “Circulating Ceramides as Biomarkers of Cardiovascular Disease: Evidence From Phenotypic and Genomic Studies.” Atherosclerosis 327: 18–30. 34004484 10.1016/j.atherosclerosis.2021.04.021

[cph470053-bib-0034] Metwally, E. , A. Sanchez Solano , B. Lavanderos , et al. 2024. “Mitochondrial Ca^2+^−Coupled Generation of Reactive Oxygen Species, Peroxynitrite Formation, and Endothelial Dysfunction in Cantú Syndrome.” JCI Insight 9: e176212. 39088268 10.1172/jci.insight.176212 PMC11385080

[cph470053-bib-0035] Mirza, I. , M. Haloul , C. Hassan , et al. 2023. “Adiposomes From Obese‐Diabetic Individuals Promote Endothelial Dysfunction and Loss of Surface Caveolae.” Cells 12: 2453. 37887297 10.3390/cells12202453 PMC10605845

[cph470053-bib-0036] Mirza, I. , C. Hassan , M. Masrur , F. M. Bianco , M. M. Ali , and A. M. Mahmoud . 2022. “The Role of Adipocyte‐Derived Extracellular Vesicles in Diabetes‐Associated Endothelial Dysfunction.” FASEB Journal 36: 36.S1.R2259. 10.1096/fasebj.2022.36.S1.R2259.

[cph470053-bib-0037] Mirza, I. , D. Naquiallah , C. Hassan , et al. 2022. “Abstract 13966: The Effect of Diabetes‐Associated Adiposomes in Promoting Caveolar Loss and Endothelial Dysfunction.” Circulation 146: A13966.

[cph470053-bib-0038] Moral‐Sanz, J. , T. Gonzalez , C. Menendez , et al. 2011. “Ceramide Inhibits Kv Currents and Contributes to Tp‐Receptor‐Induced Vasoconstriction in Rat and Human Pulmonary Arteries.” American Journal of Physiology. Cell Physiology 301: C186–C194. 21490312 10.1152/ajpcell.00243.2010

[cph470053-bib-0039] Nichols, C. G. , G. K. Singh , and D. K. Grange . 2013. “K_ATP_ Channels and Cardiovascular Disease.” Circulation Research 112: 1059–1072. 23538276 10.1161/CIRCRESAHA.112.300514 PMC3660033

[cph470053-bib-0040] Noblet, J. N. , M. K. Owen , A. G. Goodwill , D. J. Sassoon , and J. D. Tune . 2015. “Lean and Obese Coronary Perivascular Adipose Tissue Impairs Vasodilation via Differential Inhibition of Vascular Smooth Muscle K^+^ Channels.” Arteriosclerosis, Thrombosis, and Vascular Biology 35: 1393–1400. 25838427 10.1161/ATVBAHA.115.305500 PMC4441615

[cph470053-bib-0041] Okunogbe, A. , R. Nugent , G. Spencer , J. Powis , J. Ralston , and J. Wilding . 2022. “Economic Impacts of Overweight and Obesity: Current and Future Estimates for 161 Countries.” BMJ Global Health 7: e009773. 10.1136/bmjgh-2022-009773 PMC9494015 36130777

[cph470053-bib-0042] Olson, T. M. , and A. Terzic . 2010. “Human k(Atp) Channelopathies: Diseases of Metabolic Homeostasis.” Pflügers Archiv 460: 295–306. 20033705 10.1007/s00424-009-0771-y PMC2883927

[cph470053-bib-0043] Ottolini, M. , K. Hong , E. L. Cope , et al. 2020. “Local Peroxynitrite Impairs Endothelial Transient Receptor Potential Vanilloid 4 Channels and Elevates Blood Pressure in Obesity.” Circulation 141: 1318–1333. 32008372 10.1161/CIRCULATIONAHA.119.043385 PMC7195859

[cph470053-bib-0044] Piccoli, M. , F. Cirillo , A. Ghiroldi , et al. 2023. “Sphingolipids and Atherosclerosis: The Dual Role of Ceramide and Sphingosine‐1‐Phosphate.” Antioxidants 12: 143. 36671005 10.3390/antiox12010143 PMC9855164

[cph470053-bib-0045] Powell‐Wiley, T. M. , P. Poirier , L. E. Burke , et al. 2021. “Obesity and Cardiovascular Disease: A Scientific Statement From the American Heart Association.” Circulation 143: e984–e1010. 33882682 10.1161/CIR.0000000000000973 PMC8493650

[cph470053-bib-0046] Sakers, A. , M. K. De Siqueira , P. Seale , and C. J. Villanueva . 2022. “Adipose‐Tissue Plasticity in Health and Disease.” Cell 185: 419–446. 35120662 10.1016/j.cell.2021.12.016 PMC11152570

[cph470053-bib-0047] SenthilKumar, G. , Z. Zirgibel , K. E. Cohen , et al. 2024. “Ying and Yang of Ceramide in the Vascular Endothelium.” Arteriosclerosis, Thrombosis, and Vascular Biology 44: 1725–1736. 38899471 10.1161/ATVBAHA.124.321158 PMC11269027

[cph470053-bib-0048] Shalaby, Y. M. , M. M. Nakhal , B. Afandi , et al. 2025. “Impact of Sodium‐Glucose Cotransporter‐2 Inhibitors on Aging Biomarkers and Plasma Ceramide Levels in Type 2 Diabetes: Beyond Glycemic Control.” Annals of Medicine 57: 2496795. 40289660 10.1080/07853890.2025.2496795 PMC12039402

[cph470053-bib-0049] Shi, J. , Y. Yang , A. Cheng , G. Xu , and F. He . 2020. “Metabolism of Vascular Smooth Muscle Cells in Vascular Diseases.” American Journal of Physiology. Heart and Circulatory Physiology 319: H613–H631. 32762559 10.1152/ajpheart.00220.2020

[cph470053-bib-0050] Solano, A. S. , B. Lavanderos , E. Metwally , and S. Earley . 2024. “Transient Receptor Potential Channels in Vascular Mechanotransduction.” American Journal of Hypertension 38: 151–160. 10.1093/ajh/hpae134 PMC13368814 39579078

[cph470053-bib-0051] Stapleton, P. A. , M. E. James , A. G. Goodwill , and J. C. Frisbee . 2008. “Obesity and Vascular Dysfunction.” Pathophysiology 15: 79–89. 18571908 10.1016/j.pathophys.2008.04.007 PMC2593649

[cph470053-bib-0052] Teramoto, N. 2006. “Physiological Roles of Atp‐Sensitive K^+^ Channels in Smooth Muscle.” Journal of Physiology 572: 617–624. 16484295 10.1113/jphysiol.2006.105973 PMC1779997

[cph470053-bib-0053] Valenzuela, P. L. , P. Carrera‐Bastos , A. Castillo‐García , D. E. Lieberman , A. Santos‐Lozano , and A. Lucia . 2023. “Obesity and the Risk of Cardiometabolic Diseases.” Nature Reviews. Cardiology 20: 475–494. 36927772 10.1038/s41569-023-00847-5

[cph470053-bib-0054] Van Gaal, L. F. , I. L. Mertens , and C. E. De Block . 2006. “Mechanisms Linking Obesity With Cardiovascular Disease.” Nature 444: 875–880. 17167476 10.1038/nature05487

[cph470053-bib-0055] Wang, S. , Z. Jin , B. Wu , A. J. Morris , and P. Deng . 2025. “Role of Dietary and Nutritional Interventions in Ceramide‐Associated Diseases.” Journal of Lipid Research 66: 100726. 39667580 10.1016/j.jlr.2024.100726 PMC11754522

[cph470053-bib-0056] Wenceslau, C. F. , C. G. McCarthy , S. Earley , et al. 2021. “Guidelines for the Measurement of Vascular Function and Structure in Isolated Arteries and Veins.” American Journal of Physiology. Heart and Circulatory Physiology 321: H77–h111. 33989082 10.1152/ajpheart.01021.2020 PMC8321813

[cph470053-bib-0057] Wretlind, A. , V. R. Curovic , A. de Zawadzki , et al. 2023. “Ceramides Are Decreased After Liraglutide Treatment in People With Type 2 Diabetes: A Post Hoc Analysis of Two Randomized Clinical Trials.” Lipids in Health and Disease 22: 160. 37752566 10.1186/s12944-023-01922-z PMC10521385

[cph470053-bib-0058] Zheng, T. , W. Li , J. Wang , B. T. Altura , and B. M. Altura . 2000. “Sphingomyelinase and Ceramide Analogs Induce Contraction and Rises in [Ca^2+^]i in Canine Cerebral Vascular Muscle.” American Journal of Physiology. Heart and Circulatory Physiology 278: H1421–H1428. 10775118 10.1152/ajpheart.2000.278.5.H1421

[cph470053-bib-0059] Zhou, Y. , H. Li , and N. Xia . 2021. “The Interplay Between Adipose Tissue and Vasculature: Role of Oxidative Stress in Obesity.” Frontiers in Cardiovascular Medicine 8: 650214. 33748199 10.3389/fcvm.2021.650214 PMC7969519

[cph470053-bib-0060] Zhu, Y. , Y. Chu , S. Wang , et al. 2023. “Vascular Smooth Muscle trpv4 (Transient Receptor Potential Vanilloid Family Member 4) Channels Regulate Vasoconstriction and Blood Pressure in Obesity.” Hypertension 80: 757–770. 36794584 10.1161/HYPERTENSIONAHA.122.20109

