# Supplementary material for: Obesity‐Associated Adiposomes Promote Vascular Smooth Muscle Cell Hypercontractility
Source: Compr Physiol. 2025 Sep 18;15(5):e70053. doi: 10.1002/cph4.70053 (PMC12446896; doi:10.1002/cph4.70053)
Supplement: Supplementary file 1 — Figure S1: Small skeletal muscle arterioles from DIO mice are hypercontractile. (A) Isolation of a Small skeletal muscle arterioles (arrow) from mice. (B, C) Representative tracings showing changes in the intraluminal diameter of isolated skeletal muscle arterioles from SD and DIO mice. (D) Summary data showing the percentage of myogenic tone in skeletal muscle arterioles from SD and DIO mice. Data are presented as mean ± SEM, n = 6 vessels from 3 mice; *p < 0.05, two‐way ANOVA with Šidák's multiple comparisons test. (E, F) Summary data illustrating the passive inner diameter (μm) and distensibility coefficient of isolated skeletal muscle arterioles. Data are presented as mean ± SEM, n = 6 vessels from 3 mice per group, two‐way ANOVA followed by Šidák's multiple comparisons test, ns: not significant, p ≥ 0.05. (G) Summary data show the constriction of isolated skeletal muscle arterioles from the indicated mice in response to 60 mM KCl. Data are presented as mean ± SEM, n = 6 arteries, unpaired Student's t test, ns: not significant; p ≥ 0.05. Figure S2: Adiposomes exert their effects on vSMCs independently of endothelial cells. (A, B) Summary data showing the percentage of myogenic tone in mesenteric arterioles isolated from SD mice. Arterioles were incubated with adiposomes derived from DIO mice, either alone or in combination with the nitric oxide donor sodium nitroprusside (SNP) (10 μM) (A) or following endothelial denudation by gentle air perfusion (B). Data are presented as mean ± SEM; *p < 0.05, two‐way ANOVA with Šidák's multiple comparisons test (n = 3 vessels from 3 mice). Figure S3: Adiposome uptake in intact vessels. (A–C) Representative images of whole‐mount mesenteric blood vessels treated with adiposomes isolated from either SD or DIO mice. Adiposome uptake is visualized using BODIPY‐labeled particles (red, arrows). vSMCs are identified by α‐smooth muscle actin (α‐SMA, green), and nuclei are counterstained with DAPI (blue). Scale bar = 50 μm. Figure S4: [file CPH4-15-e70053-s009.docx]

**Supplementary Figures**


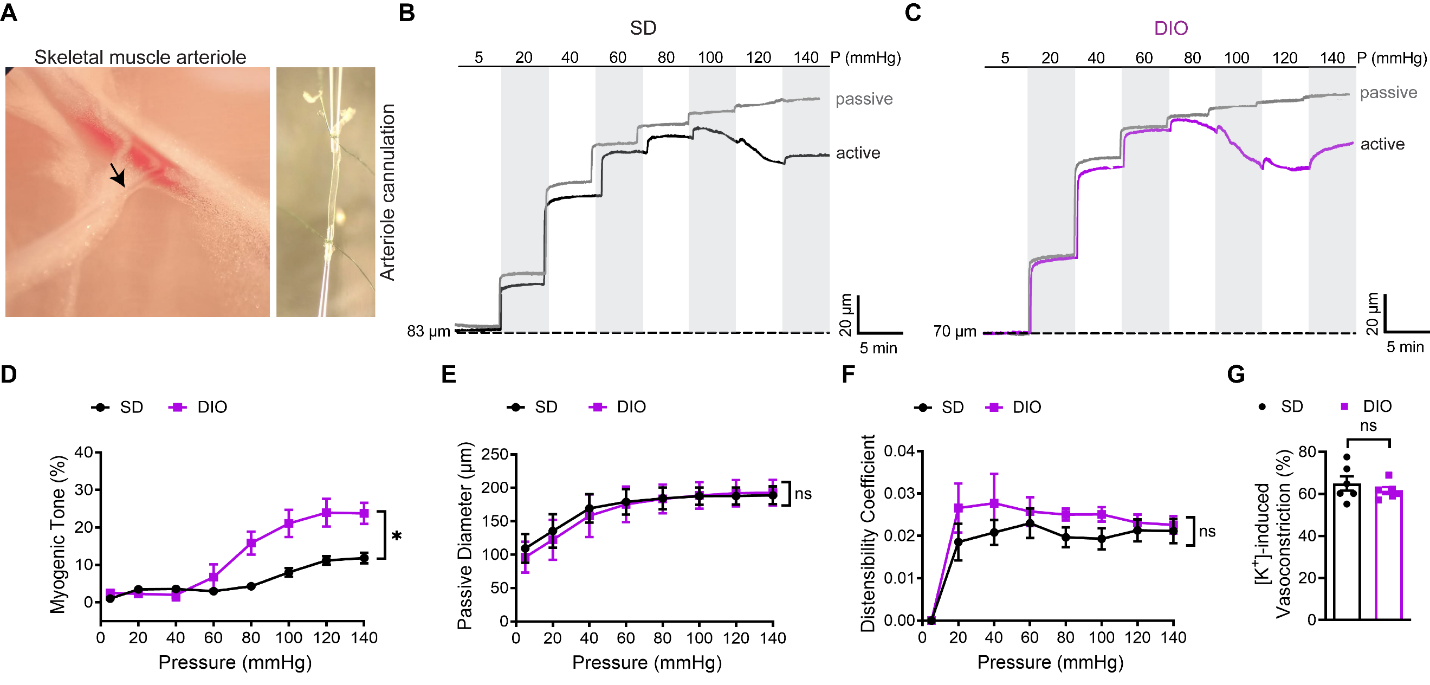


**Figure S1. Small skeletal muscle arterioles from DIO mice are hypercontractile. A,** Isolation of a Small skeletal muscle arterioles (arrow) from mice. **B and C,** Representative tracings showing changes in the intraluminal diameter of isolated skeletal muscle arterioles from SD and DIO mice. **D,** Summary data showing the percentage of myogenic tone in skeletal muscle arterioles from SD and DIO mice. Data are presented as means ± SEM, n = 6 vessels from 3 mice; **p* < 0.05, two-way ANOVA with Šidák's multiple comparisons test. **E and F,** Summary data illustrating the passive inner diameter (µm) and distensibility coefficient of isolated skeletal muscle arterioles. Data are presented as means ± SEM, n = 6 vessels from 3 mice per group, two-way ANOVA followed by Šidák's multiple comparisons test, ns: not significant, *p* ≥ 0.05. **G,** Summary data show the constriction of isolated skeletal muscle arterioles from the indicated mice in response to 60 mM KCl. Data are presented as means ± SEM, n = 6 arteries, unpaired student's *t*-test, ns: not significant; p ≥ 0.05.


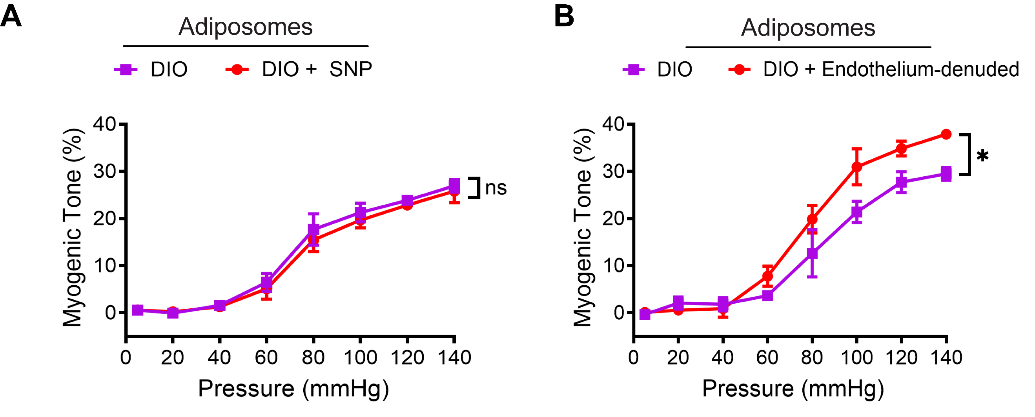


**Figure S2.** **Adiposomes exert their effects on vSMCs independently of endothelial cells.** (**A–B**) Summary data showing the percentage of myogenic tone in mesenteric arterioles isolated from SD mice. Arterioles were incubated with adiposomes derived from DIO mice, either alone or in combination with the nitric oxide donor sodium nitroprusside (SNP) (10 µM) (**A**) or following endothelial denudation by gentle air perfusion (**B**). Data are presented as mean ± SEM; *p < 0.05, two-way ANOVA with Šidák’s multiple comparisons test (n = 3 vessels from 3 mice).


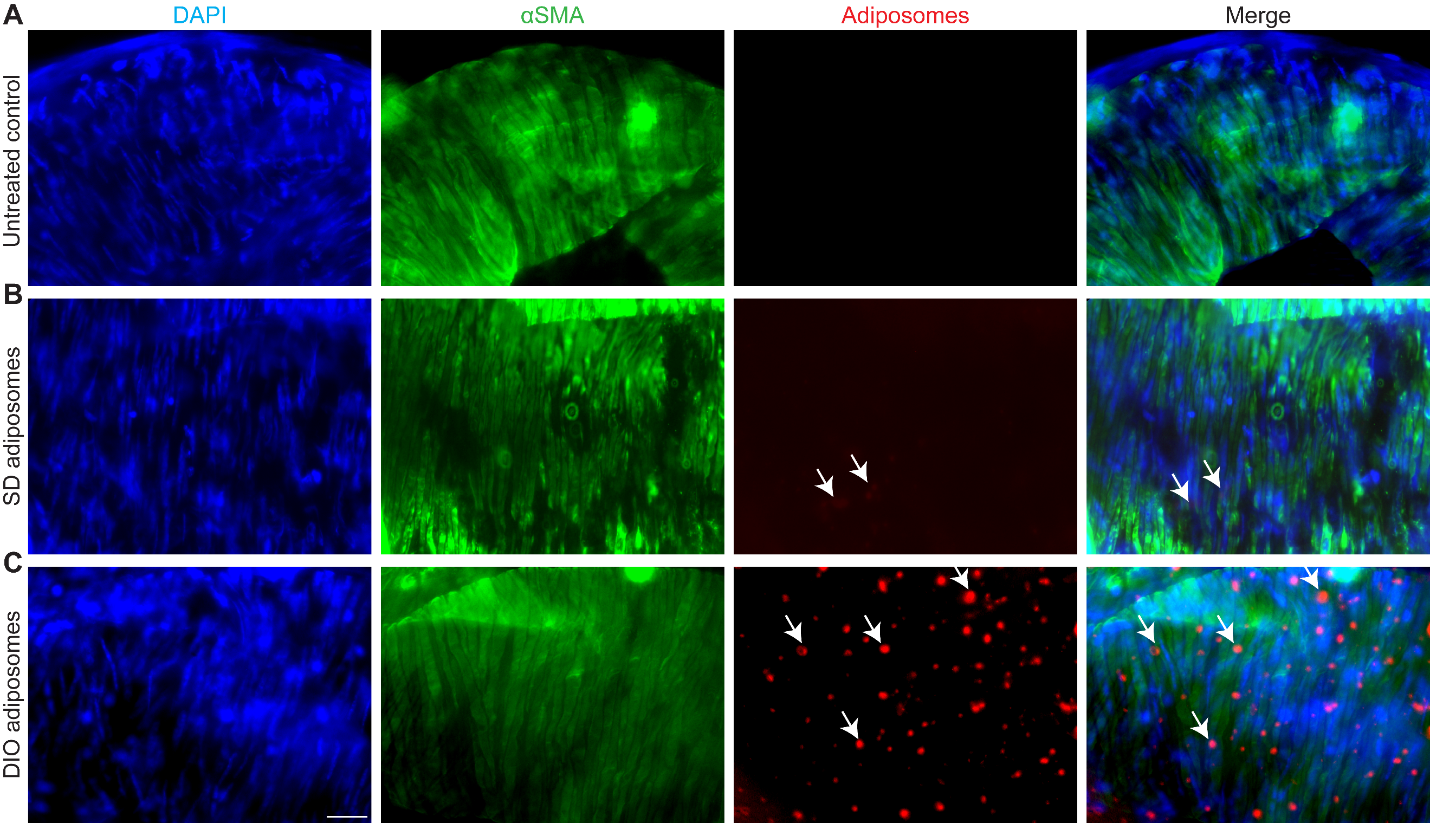


**Figure S3. Adiposome uptake in intact vessels.** (**A–C**) Representative images of whole-mount mesenteric blood vessels treated with adiposomes isolated from either SD or DIO mice. Adiposome uptake is visualized using BODIPY-labeled particles (red, arrows). vSMCs are identified by α-smooth muscle actin (α-SMA, green), and nuclei are counterstained with DAPI (blue). Scale bar = 50 µm.


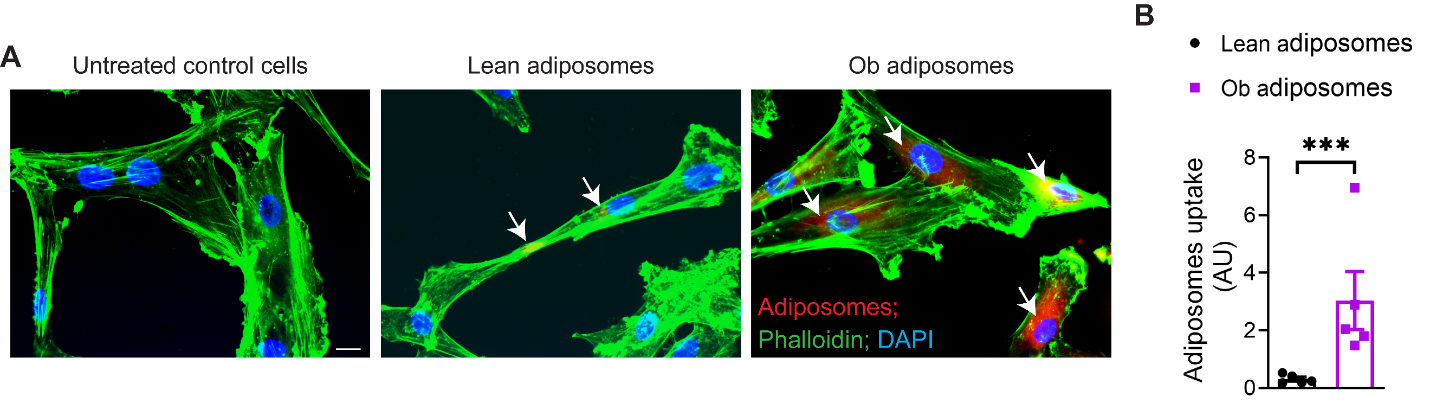
**Figure S4. Adiposome uptake with cultured vSMCs. A,** Representative image of adiposome uptake labeled with BODIPY dye (red, arrows) by vSMCs (cell membrane labeled green [phalloidin] and nuclei labeled blue [DAPI]). Scale bar = 50 µm. **B,** Summary data showing red fluorescent signal intensity expressed in arbitrary units (AU) in response to adiposome treatment isolated from lean and obese individuals. Data are presented as means ± SEM, n = 5 cells per group (cells were imaged from independent preparations), ****p* < 0.001, unpaired Student's *t*-test).


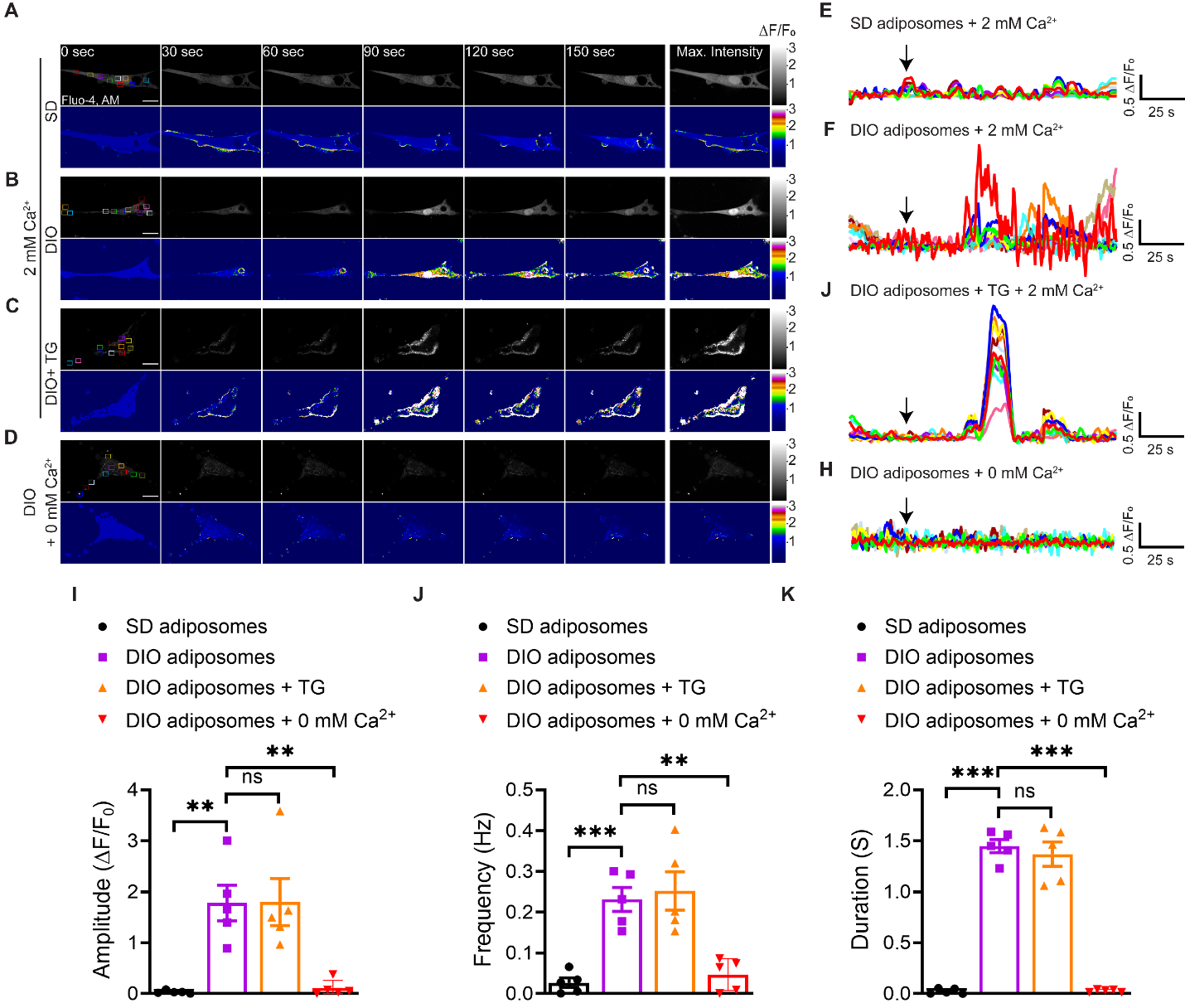


**Figure S5. DIO mice adiposomes increase Ca²⁺ influx in cultured vSMCs. A–D,** Representative grayscale and pseudocolored images of cultured vSMCs treated with adiposomes isolated from SD and DIO mice under different conditions. Cells were stained with Fluo-4 AM. Recordings were initially made under baseline conditions for 30 seconds, followed by exposure to adiposomes in a Ca²⁺-containing solution or pretreatment with thapsigargin (TG) and a Ca²⁺-free solution for 120 seconds. Colored boxes highlight ROIs with active Ca²⁺ signals. Scale bar = 50 µm**. E–H,** Representative ΔF/F₀ vs. time plots showing Ca²⁺ traces from multiple ROIs under the indicated conditions. **I–K**, Summary data showing the amplitude (ΔF/F₀), frequency (Hz), and signal duration in SMCs. Data are presented as means ± SEM, n = 5 cells per group (cells were imaged from independent preparations); ***p* < 0.01; ****p* < 0.001, two-way ANOVA with Tukey's multiple comparisons test; ns: not significant, p ≥ 0.05.


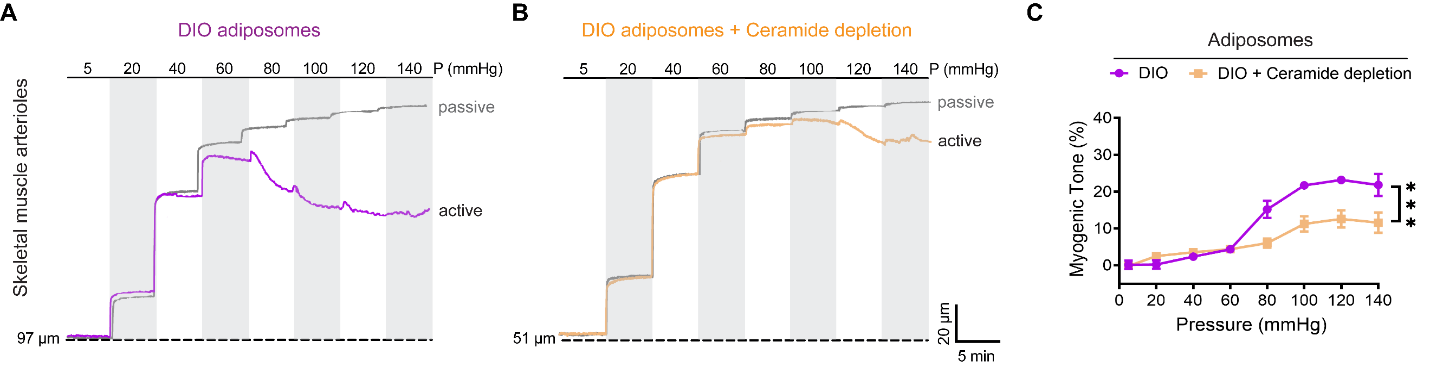


**Figure S6. Ceramide depletion attenuates adiposome-mediated hypercontractility in skeletal muscle arterioles**. **A and B**,. Representative tracings showing changes in the intraluminal diameter of isolated skeletal muscle arterioles from SD mice following co-incubation with adiposomes or ceramide-depleted adiposomes isolated from DIO mice. **C**, Summary data quantifying the percentage of myogenic tone. Data are expressed as means ± SEM, n = 3 vessels from 3 mice; ****p* < 0.001, two-way ANOVA with Šidák's multiple comparisons test.

**Supplementary Videos**

**Video S1.** Ca²⁺ signal in isolated native vSMCs from a lean individual, recorded in the presence of ACh (5 µM).

**Video S2.** Spontaneous and ACh-evoked Ca²⁺ events in isolated native vSMCs isolated from an obese individual. Recordings show intracellular Ca²⁺ dynamics following stimulation with ACh (5 µM).

**Video S3.** Pretreatment with thapsigargin (TG, 2 µM) did not abolish spontaneous ACh-evoked Ca²⁺ events in isolated native vSMCs isolated from an obese individual.

**Video S4.** Ca²⁺ signaling in cultured vSMCs following exposure to adiposomes derived from a lean individual in the presence of 2 mM extracellular Ca²⁺.

**Video S5.** Ca²⁺ signaling in cultured vSMCs following exposure to adiposomes derived from an obese individual in the presence of 2 mM extracellular Ca²⁺.

**Video S6.** Ca²⁺ signaling in cultured vSMCs following exposure to adiposomes derived from an obese individual in the presence of 0 mM extracellular Ca²⁺.

**Video S7.** Ca²⁺ signaling in cultured vSMCs following exposure to adiposomes derived from SD mice in the presence of 2 mM extracellular Ca²⁺.

**Video S8.** Ca²⁺ signaling in cultured vSMCs following exposure to adiposomes derived from DIO mice in the presence of 2 mM extracellular Ca²⁺.

**Video S9.** Ca²⁺ signaling in cultured vSMCs following exposure to adiposomes derived from DIO mice in the presence of 2 mM extracellular Ca²⁺ and Pretreatment with thapsigargin (TG, 2 µM).

**Video S10.** Ca²⁺ signaling in cultured vSMCs following exposure to adiposomes derived from DIO mice in the presence of 0 mM extracellular Ca²⁺.

**Supplementary Tables**

**Table S1.** Clinical characteristics of lean vs. obese participants

| Parameter | Lean (12) | Obese (25) | p-value | Significance |
| --- | --- | --- | --- | --- |
| Age, years | 34.8 ± 5.2 | 35.8 ± 9.18 | 0.701 | ns |
| BMI, kg/m² | 22.4 ± 1.2 | 51.08 ± 5.99 | <0.001 | *** |
| Body fat, % | 20.5 ± 3.5 | 56.1 ± 2.8 | <0.001 | *** |
| Visceral fat mass, kg | 0.5 ± 0.2 | 4.2 ± 0.2 | <0.001 | *** |
| Waist circumference, cm | 90.5 ± 5.1 | 130.2 ± 8.3 | <0.001 | *** |
| Fasting glucose, mg/dL | 94.1 ± 11.3 | 104.08 ± 28.7 | 0.198 | ns |
| HbA1c, % | 5.4 ± 0.2 | 5.53 ± 0.627 | 0.496 | ns |
| Total cholesterol, mg/dL | 148.5 ± 31.1 | 169.35 ±30.76 | 0.043 | * |
| LDL, mg/dL | 83.1 ± 18.2 | 96.95 ± 25.11 | 0.049 | * |
| HDL, mg/dL | 55.1 ± 16.2 | 50.25 ± 10.20 | 0.301 | ns |
| Triglycerides, mg/dL | 88.9 ± 25.0 | 120.65 ±42.46 | 0.012 | ** |
| Total Hemoglobin, g/dL | 13.5 ± 1.5 | 12.3 ± 1.1 | 0.017 | * |
| Heart rate, BPM | 76 ± 12 | 81 ± 13 | 0.238 | ns |
| Systolic BP, mmHg | 119 ± 12 | 132 ± 17 | 0.011 | ** |
| Diastolic BP, mmHg | 75 ± 9 | 79 ± 11 | 0.241 | ns |
